# Discovery and Characterization of the Metallopterin-Dependent Ergothioneine Synthase from *Caldithrix abyssi*

**DOI:** 10.1021/jacsau.2c00365

**Published:** 2022-08-16

**Authors:** Mariia A. Beliaeva, Florian P. Seebeck

**Affiliations:** † Department of Chemistry, University of Basel, Mattenstrasse 24a, 4002 Basel, Switzerland; ‡ Molecular Systems Engineering, National Competence Center in Research (NCCR), 4058 Basel, Switzerland

**Keywords:** ergothioneine, molybdopterin, sulfur transfer, cysteine desulfurase

## Abstract

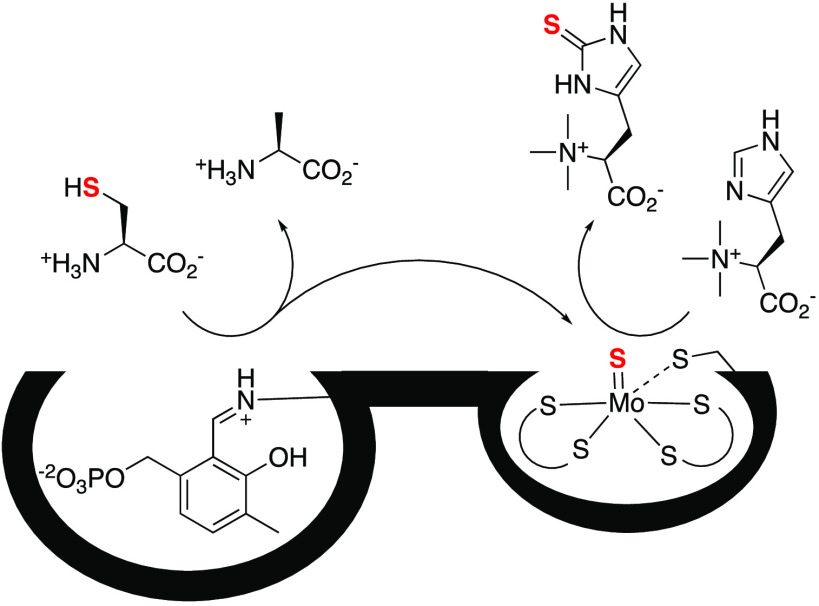

Ergothioneine is a histidine derivative with a 2-mercaptoimidazole side chain and a trimethylated α-amino group. Although the physiological function of this natural product is not yet understood, the facts that many bacteria, some archaea, and most fungi produce ergothioneine and that plants and animals have specific mechanisms to absorb and distribute ergothioneine in specific tissues suggest a fundamental role in cellular life. The observation that ergothioneine biosynthesis has emerged multiple times in molecular evolution points to the same conclusion. Aerobic bacteria and fungi attach sulfur to the imidazole ring of trimethylhistidine *via* an O_2_-dependent reaction that is catalyzed by a mononuclear non-heme iron enzyme. Green sulfur bacteria and archaea use a rhodanese-like sulfur transferase to attach sulfur *via* oxidative polar substitution. In this report, we describe a third unrelated class of enzymes that catalyze sulfur transfer in ergothioneine production. The metallopterin-dependent ergothioneine synthase from *Caldithrix abyssi* contains an N-terminal module that is related to the tungsten-dependent acetylene hydratase and a C-terminal domain that is a functional cysteine desulfurase. The two modules cooperate to transfer sulfur from cysteine onto trimethylhistidine. Inactivation of the C-terminal desulfurase blocks ergothioneine production but maintains the ability of the metallopterin to exchange sulfur between ergothioneine and trimethylhistidine. Homologous bifunctional enzymes are encoded exclusively in anaerobic bacterial and archaeal species.

## Introduction

**Figure 1:**
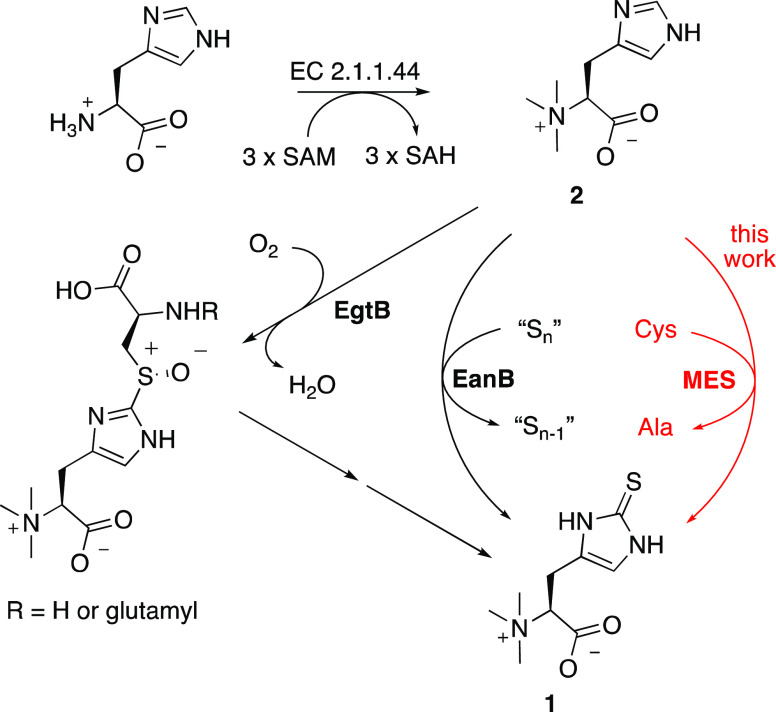
Three biosynthetic strategies for the sulfurization of *N*α-trimethylhistidine (TMH) that emerged through convergent evolution. (i) O_2_-dependent sulfurization by sulfoxide synthase EgtB; (ii) O_2_-independent pathway catalyzed by ergothioneine synthase EanB with polysulfides as a sulfur donor; (iii) O_2_-independent sulfurization by a metallopterin (MPT)-dependent ergothioneine synthase MES that uses cysteine as a sulfur donor (red).

Ergothioneine (**1**, Figure 1) is a metabolite in many bacterial, archaeal, and eukaryotic species. Despite wide distribution in higher organisms, ergothioneine is almost exclusively produced by microorganisms. Humans and other animals absorb this compound from their diet through a specific ergothioneine transporter (ETT).^1,2^ A growing body of evidence indicates that ergothioneine may protect against inflammation and reduces the risk of cardiovascular and neurological diseases.^3−5^ The remarkable redox activity of the 2-mercaptoimidazole side chain is consistent with the idea that ergothioneine might protect tissue against damage induced by UV radiation, reactive oxygen species, or redox active transition metals.^6^ However, it is not yet clear as to how these *in vitro* properties translate to protective effects *in vivo*.^7,8^

Ergothioneine is not only ubiquitous but also has been “invented” multiple times in the course of natural evolution. The most common pathway occurs in fungi, actinobacteria, cyanobacteria, and many proteobacteria.^9−12^ These organisms are indeed often exposed to light, drought, and oxygen, which is consistent with the idea that ergothioneine could serve as an antioxidant. In this pathway, first an *S*-adenosylmethionine-dependent methyltransferase (EC 2.1.1.44) converts histidine to *N*α-trimethylhistidine (**2**, TMH).^13^ In a second step, an iron-dependent sulfoxide synthase (EgtB or Egt1, EC 1.14.99.50/52) catalyzes O_2_-dependent coupling of a cysteine or γ-glutamylcysteine to carbon 2 of the imidazole ring of TMH (Figure 1).^14−16^ Subsequent steps trim the resulting sulfoxide intermediate to ergothioneine. A similar pathway emerged by convergent evolution in a small group of cyanobacteria that recruited a sulfoxide synthase from a different process to make ergothioneine.^17^ An alternative way that involves different chemistry is catalyzed by an O_2_-independent ergothioneine synthase (EanB) from the green sulfur bacterium *Chlorobium limicola*.^18^ EanB is a rhodanese-like enzyme that transfers sulfur from polysulfides to TMH by nucleophilic aromatic substitution,^19−21^ or, as suggested by others, through a carbene-type mechanism.^22^ Because most EanB homologues are encoded in anaerobic bacteria and archaea, the discovery of this pathway raised questions as to whether ergothioneine may also serve functions under anoxic conditions and whether this compound may be of ancient origin.

**Figure 2:**
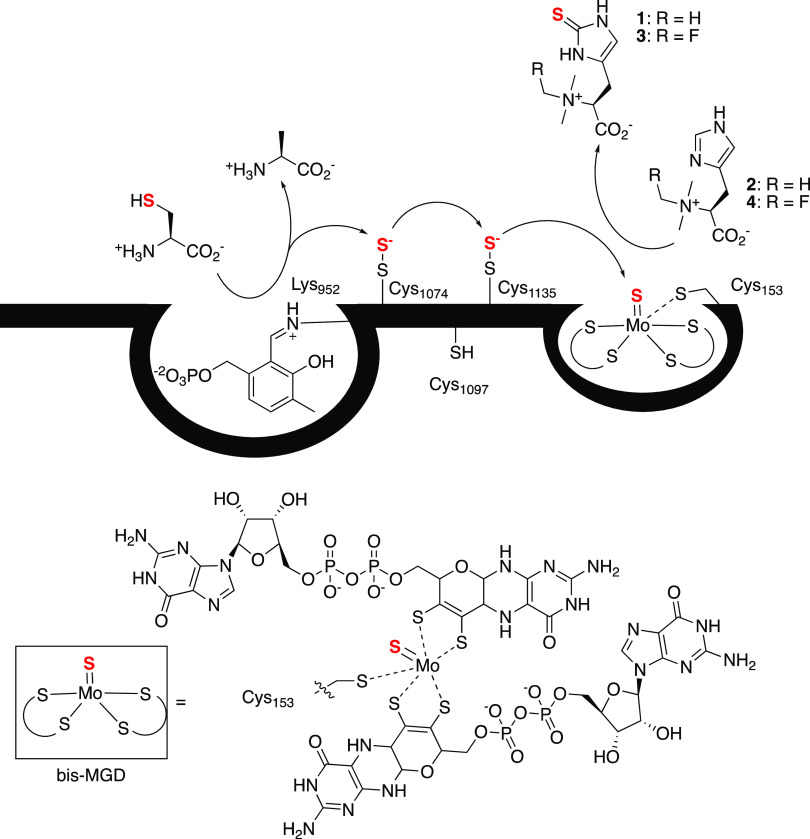
Top: schematic structure of MES. The C-terminal cysteine desulfurase domain extracts sulfur from l-cysteine. This sulfur atom is transported to the N-terminal metallopterin-binding module that mediates oxidative sulfurization of TMH. The intramolecular transport of the sulfur atom may occur *via* transient formation of persulfides on Cys1074 and Cys1135 (persulfide relay).^36,37^ Mutation of Lys952, Cys1074, Cys1135, or Cys153 eliminates all measurable ergothioneine biosynthesis in recombinant *Escherichia coli*. The mechanism by which desulfurases convert cysteine to alanine has been discussed previously.^38^ A possible mechanism by which the MPT-binding domain introduces sulfur into TMH is shown in Figure 5. Bottom: proposed structure of the bis-MGD cofactor of MES. All members of the DMSO reductase (DMSOR) family bind similar cofactors with either terminal oxo or sulfido ligands and with Ser, Asp, or Cys as a single protein-derived metal ligand.^23,26^

This report adds further evidence to these notions. We describe a third unrelated class of enzymes that catalyzes ergothioneine production. These proteins consist of an N-terminal metallopterin-binding domain (MPT) and a C-terminal pyridoxyl-5-phosphate-dependent (PLP) cysteine desulfurase (Figure 2). Our observations suggest that the two modules cooperate to transfer a sulfur atom from l-cysteine to TMH. Among the diverse family of mononuclear molybdenum- and tungsten-dependent enzymes,^23−26^ the metallopterin-dependent ergothioneine synthase (MES) is the first example that catalyzes carbon–sulfur bond formation. Homologous enzymes occur in a variety of strictly anaerobic organisms, including *Caldithrix abyssi* and other bacteria that were isolated from deep-ocean hydrothermal vents.^27^

## Results and Discussion

### Identification of an MPT-Dependent Ergothioneine Synthase

Three-fold methylation at the α-amino group of histidine is the first step in ergothioneine biosynthesis in any pathway (Figure 1). This activity was discovered in cell-free lysates from *Neurospora crassa*.^28^ The gene coding for an enzyme (EgtD) with this specific activity was identified in the genome of *Mycobacterium smegmatis*.^10^ The crystal structure of EgtD in the complex with substrates, intermediates, and products revealed sequence motifs that allow reliable prediction of histidine-specific methyltransferases based on primary sequences (Figure S1).^13,29−31^ Most members of this enzyme family (IPR035094) occur in species that also contain EgtB-like sulfoxide synthases or EanB-like ergothioneine synthases. In addition, we also identified approximately 80 genomes (Table S1) coding for histidine-specific methyltransferases but without any discernable candidate for the TMH sulfurizing enzyme. Instead, in 55 of these genomes, the methyltransferases are co-encoded with a putative PLP-dependent cysteine desulfurase fused to an MPT-binding enzyme. As the subsequent analysis will show, these enzymes are MESs.

The N-terminal MPT-binding module (residues 1–718) of MES from *C. abyssi* (*Ca*MES) shares low but significant sequence similarity to the tungsten-dependent acetylene hydratase (ACH) from *Syntrophotalea acetylenica* (27%, PDB code: 2E7Z)^32^ and to the molybdenum-dependent DMSO reductase (DMSOR) from *Cereibacter sphaeroides* (27%, 1EU1).^33^ These enzymes have a bis-metallopterin guanine dinucleotide cofactor (bis-MGD, Figure 2) that forms a single coordination bond between the metal center and a Cys (ACH) or a Ser (DMSOR) residue. α-Fold modeling predicts that the MPT-binding module of *Ca*MES adopts a very similar overall fold.^34^ Furthermore, sequence alignment (Figure S2) suggests that the residues that make direct contacts with the bis-MGD cofactor in ACH and DMSOR are largely conserved (Figure S2), suggesting that *Ca*MES likely binds the same cofactor and that the metal is coordinated by a Cys residue (Cys153, Figure 2). Conservation of four Cys residues at the N-terminus (Cys14, Cys17, Cys21, and Cys48) indicates that MES contains a similar iron/sulfur (Fe_4_S_4_) cluster as ACH. The C-terminal module (residues 747–1136) of MES resembles bacterial cysteine desulfurases, such as NifS from *Helicobacter pylori* (44%, PDB: 5WT2) or IscS from *E. coli* (44%, 1P3W). Members of the class V aminotransferase protein family (IPR000192) form obligatory dimers because their two symmetry-related active sites consist of residues from both chains.^35^ Therefore, we can expect that the MES is also dimeric.

### Recombinant MES Produces Ergothioneine in *E. coli*

Despite testing several conditions for cell culturing, we were unable to purify full-length MES from recombinant *E. coli*. Therefore, we examined the activity of this enzyme *in vivo*. To quantify the concentration of intracellular ergothioneine in *E. coli*, we established a protocol for cell lysate analysis by way of high-resolution electron-spray mass spectrometry (HR-ESI-MS) using heavy isotopologues of TMH and ergothioneine for calibration (Supporting Information).^20^ This method allows the detection of the two compounds at cellular concentrations as low as 20 nM (Figure S6). With this assay, we could establish that untransformed *E. coli* grown in either Luria–Bertani broth (LB) or chemically defined medium (CD medium: M9 salts, amino acids, glucose, vitamins) do not contain ergothioneine above this detection limit (Table S4). In contrast, TMH could be detected in cells grown in LB medium (110 nM) and in CD medium (70 nM) likely because the growth media also contain this compound at a level near the detection limit (LB: 330 nM, CD: <20).

We transformed a strain derived from *E. coli* K-12 (*E. coli* Δmtn)^39,40^ with pCOLADuet-1 vectors each containing a codon-optimized gene for a MES homologue from *Candidatus Abyssubacteria* bacterium (*Ab*MES),^41^*C. abyssi* (*Ca*MES),^27^ and *Euryarchaeota archaeon* ADurb.Bin009 (*Ea*MES) or from a hydrothermal vent metagenome (*hv*MES). The transformed *E. coli* cells were grown in CD medium (Table S2) at 37 °C to an optical density (OD_600_) of 0.5. The cultures were supplemented with 0.1 mM isopropyl β-d-1-thiogalactopyranoside (IPTG), 1 mM TMH, and 10 μM Na_2_MoO_4_ or Na_2_WO_4_ and incubated further for 20 h at 25 °C (Table 1). Quantification by high-performance liquid chromatography (HPLC) high resolution electrospray ionization mass spectrometry (ESI-HRMS) showed that all four cell lines produced ergothioneine, although with significantly different efficiencies (Table 1, entries 1 and 3–5). Cells with the gene for *Ca*MES contained the highest concentration of ergothioneine by far (Table 1, entry 1). Therefore, we focused on *Ca*MES for subsequent experiments. The data in Table 1 also reveal that the production rates are largely independent of supplemental molybdenum or tungsten. Apparently, even the chemically defined medium contained sufficient levels of one of these metals. Conversely, the observation that supplementation of Mo or W did not inhibit the activity indicates that MES is active with either metal. Indeed, several members of the DMSOR family, namely, DMSOR, ACH, and trimethyl amine oxidase (TMAO), were shown to be quite promiscuous in this regard.^25,42−44^

**Table 1:** Ergothioneine Production by Recombinant MES in *E. coli*^a^

entry	enzymes	strain	+MoO_4_^2–^	+WO_4_^2–^	no metal
1	***-***	*E. coli* (Δmtn)	<20 nM	<20 nM	<20 nM
2	*Ca*MES	*E. coli* (Δmtn)	370 ± 31 μM	411 ± 146 μM	420 ± 72 μM
3	*Ca*MES	*E. coli* BL21	22 ± 2 μM	n.a.	23 ± 3 μM
4	*Ab*MES	*E. coli* (Δmtn)	0.4 ± 0.3 μM	0.4 ± 0.2 μM	0.4 μM
5	*Ea*MES	*E. coli* (Δmtn)	10 ± 8 μM	27 ± 22 μM	0.6 μM
6	*hv*MES	*E. coli* (Δmtn)	85 ± 5 μM	83 ± 3 μM	153 μM

a Cells were grown in CD medium in the presence of 1 mM TMH, 0.1 mM IPTG, and 10 μM Mo or W for 20 h at 25 °C. Cell-free lysates were cleared by centrifugation. Ergothioneine content was estimated based on an external standard (see the Supporting Information).

BL21 cells transformed with the *Ca*MES encoding vector produced 10-fold less ergothioneine (entry 3, Table 1 and Figure S7). This observation is consistent with the known difficulties of BL21 cells to produce MPT-dependent enzymes.^45,46^*Ca*MES-containing cells (*E. coli* Δmtn) grown in the presence of *N*α-methyl fluoro-TMH (**4**: F-TMH, Figure 2) instead of TMH produced *N*α-methyl fluoro ergothioneine (**3**: F-ERG, *m*/*z* calcd: 248.0864; obsd: 248.086) with yields that correlate with the concentration of F-TMH in the medium (Figure S8). Since F-TMH is an exclusively synthetic compound,^47^ we can eliminate any possibility that the detected F-ERG originates from the growth medium instead from *de novo* biosynthesis by *Ca*MES. Finally, since *Ca*ME is translated from a synthetic codon-optimized gene, we can also exclude the possibility that the foreign nucleic acid instead of the translated protein is responsible for the sulfurization of TMH in *E. coli*.

**Figure 3:**
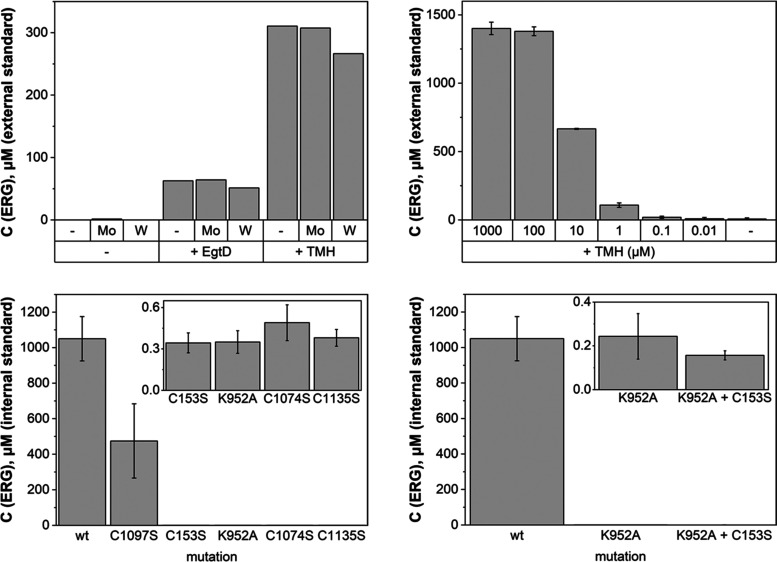
Top left: ergothioneine production by *E. coli* Δmtn cells containing *Ca*MES and EgtD or *E. coli* Δmtn cells containing *Ca*MES grown with supplemental TMH. Cells were grown in CD medium additionally supplemented with 10 μM Na_2_MoO_4_ (+Mo) or Na_2_WO_4_ (+W) for 20 h at 25 °C. Ergothioneine content was estimated based on single measurements with internal standard. Top right: ergothioneine production by *Ca*MES in *E. coli* Δmtn strain grown in CD medium supplemented with 0.01–1000 μM of TMH and/or 10 μM Na_2_MoO_4_ (+Mo) for 20 h at 25 °C. Ergothioneine content was estimated based on an external standard. Bottom: ergothioneine production by *Ca*MES mutants (right) and two-plasmid construct (left) in *E. coli* Δmtn strain grown in the CD medium supplemented with 100 μM of TMH and 10 μM Na_2_MoO_4_ for 20 h at 25 °C. Ergothioneine content was estimated based on internal standard. Values given with standard deviations are averages from three independent measurements.

In the next step, we examined as to whether production of ergothioneine is more efficient if TMH is produced by an intracellular histidine methyltransferase. To this end, we transformed *E. coli* Δmtn cells with two plasmids: one coding for *Ca*MES and the other coding for EgtD from *M. smegmatis* (pET19_EgtD). After cultivation in CD medium for 20 h, these cells contained approximately 200–300 μM ergothioneine. By contrast, *E. coli* Δmtn cells without EgtD produced >1 mM ergothioneine when supplemented with 1 mM TMH (Figure 3). These results suggest that an uptake from medium is a more efficient source of TMH than intracellular biosynthesis. Quantification of ergothioneine production in the presence of 0.01–1000 μM TMH showed that 100 μM already saturates the system (Figure 3, top right). These data also show that ergothioneine accumulates to concentrations that are up to100-fold higher than the concentration of TMH in the growth medium. It appears that the import of TMH across the cellular membranes—presumably by promiscuous cation transporters—is more efficient than the export of ergothioneine. However, secretion of ergothioneine by recombinant strains of *E. coli* has been reported.^48^

### Specific Transfer Path for Sulfur Atoms

After confirming that MES from several organisms mediates ergothioneine production, we turned to the question as to how these bifunctional enzymes may work. First, we confirmed the prediction that the C-terminal module of *Ca*MES (*Ca*MES_C-term_) is indeed a cysteine desulfurase (EC 2.8.1.7). This protein was produced in *E. coli* by expression of a pET28a plasmid coding for residues 747–1136 of *Ca*MES with an N-terminal His_6_-tag. Unlike the full-length protein, this fragment produced very well and could be purified to satisfactory homogeneity following standard protocols (Figure S3). A reaction containing 2 μM of this enzyme affords complete conversion of 0.5 mM cysteine to alanine in the presence of 10 mM dithiothreitol (DTT) in 50 mM phosphate buffer (pH 8.0) at 25 °C within 2 h (Figures S4 and S5). This *in vitro* activity suggests that the sulfur in MES-produced ergothioneine originates directly from l-cysteine. This result also shows that the C-terminal desulfurase of MES is stable and active in the absence of the N-terminal domain.

Previous studies on NifS- and IscS-type cysteine desulfurases have shown that the sulfur atoms from the substrate cysteines are transferred onto a cysteine residue near the active site. From this position, the sulfane sulfur is loaded onto a variety of external sulfur carriers.^38^ The C-terminal desulfurase of MES contains a structurally related cysteine (Cys1074). Two additional cysteines, Cys1097 and Cys1135, are also strictly conserved among MES homologues. We surmised that these residues may also play a role in transporting sulfur to the N-terminal MPT site. Sulfurization of molybdenum cofactors by cysteine desulfurases is indeed a common theme among molybdenum-containing enzymes.^49^ For example, the chaperone XdhC from *Rhodobacter capsulatus* introduces a terminal sulfido ligand to the molybdenum center before the cofactor is inserted into xanthine dehydrogenase.^24^ The cofactor of *E. coli* formate dehydrogenase receives a sulfido prior to insertion.^50^ In *Arabidopsis thaliana*, a moco sulfurase (Aba3) transfers a sulfur atom to molybdenum as the last maturation step before the MPT cofactor is inserted into aldehyde oxidases or xanthine oxidase.^36,37^ In contrast to these documented examples, sulfurization of the cofactor in MES must occur in every catalytic cycle and most likely does not involve extraction and reinsertion of the cofactor by a chaperone.

As a first test of the idea that cysteine-derived sulfur atoms may migrate from the C- to the N-terminal module *via* a persulfide relay, we examined the ergothioneine productivity of *Ca*MES variants lacking each one of these conserved cysteines (*Ca*MES_C1074S_, *Ca*MES_C1097S_, *Ca*MES_C1135S_). As a control, we also examined a variant with an inactivated desulfurase. This particular variant was constructed by mutating the conserved active site Lys, which forms the activating iminium bond with the PLP cofactor (*Ca*MES_K952A_, Figure 2). Quantification of ergothioneine in cells grown under standard conditions (*E. coli* Δmtn cells, CD media, 10 μM Na_2_MoO_4_, 100 μM TMH, 20 h at 25 °C) revealed that mutation of Cys1097 caused only a two-fold decrease in production compared to wild type (Figure 3). In contrast, the three remaining mutants produced 2000-fold less ergothioneine. This result suggests that (a) cysteine desulfurase activity by the C-terminal module is obligatory for ergothioneine production and (b) Cys1074 and Cys1135 are essential for the transport of sulfur to the TMH sulfurizing center (Figure 2).

**Figure 4:**
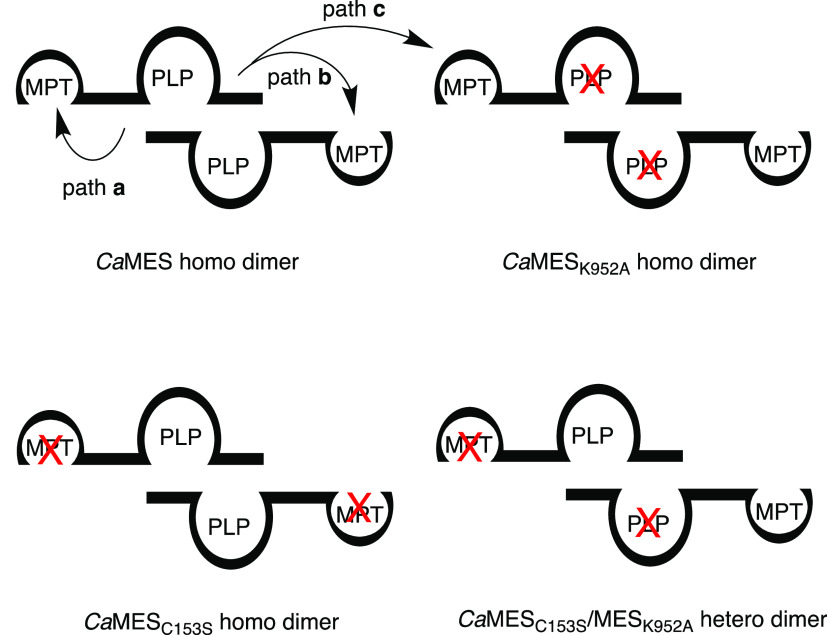
Evidence that the MPT-binding modules accept sulfur exclusively from the C-terminal desulfurase domain from the same polypeptide (path a, intramolecular), whereas intermolecular transfer (path b or c) is inefficient. Cells containing MES variants with an inactivated desulfurase module (*Ca*MES_K952A_) or an inactivated MPT-binding module (*Ca*MES_C153S_) produce no ergothioneine. Coproducing the two variants in one strain of *E. coli* gives rise to heterodimeric MES proteins (*Ca*MES_K952A_/*Ca*MES_C153S_). The observation that these heterodimers are also inactive shows that sulfur transfer between *Ca*MES polypeptides is inefficient.

We then asked whether the C-terminal desulfurase module delivers sulfur exclusively to the N-terminal module on the same polypeptide (path a, Figure 4) or to the second chain in the dimer (path b), or a different dimer (path c), or whether there are no such restrictions at all. To address this question, we examined as to whether an MES variant with an inactivated desulfurase module (*Ca*MES_K952A_) can be complemented with a variant with an inactive MPT-binding module if both variants are produced within the same strain of *E. coli* and therefore could form heterodimeric complexes. To obtain a variant with an inactive MPT-binding module, we mutated Cys153 to Ser. According to a structural model and sequence alignment (Figure S2), this conserved Cys is a direct ligand to the bis-MGD cofactor (Figure 2). Mutations of the single ligand of the MPT-cofactor usually lead to dramatic loss of activity in members of the DMSOR family.^51−53^ Consistently, cells containing the *Ca*MES_C153S_ variant produced almost no ergothioneine (Figure 3). If the desulfurase module from one polypeptide can transfer sulfur to the MPT-binding module of a different polypeptide, we would expect that cells containing genes for *Ca*MES_K952A_ and *Ca*MES_C153S_ can produce ergothioneine (Figure 4). Counter to this expectation, we found that *E. coli* Δmtn cells transformed with two plasmids (pETDuet-1*_Ca*MES_K952A_ and pET28a_*Ca*MES_C153S_) did not produce more ergothioneine than cells containing only one of the mutated genes (Figure 3). Apparently, intramolecular sulfur transfer is obligatory. Sulfurization of TMH only occurs if a functional desulfurase and a functional MPT-binding module are combined in a single polypeptide.

**Figure 5:**
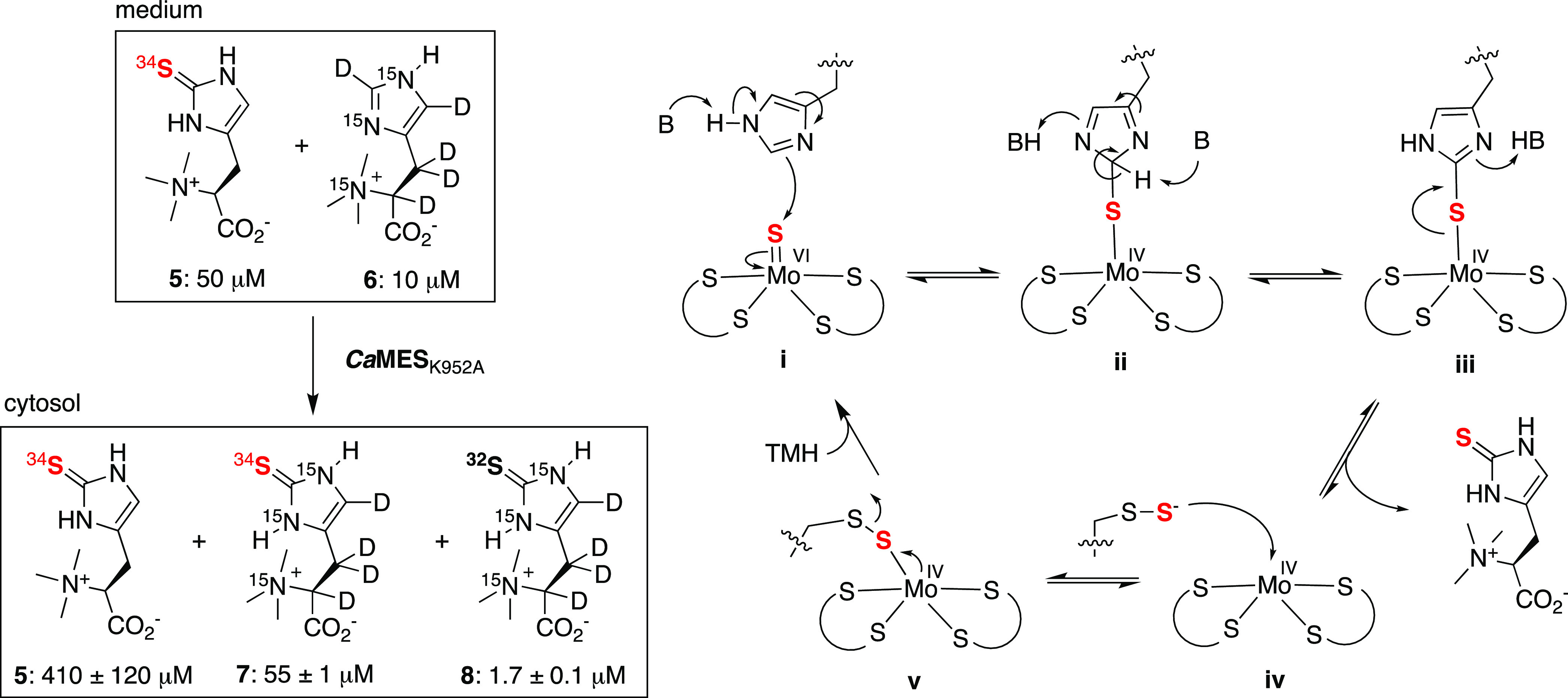
Left: *E. coli* cells containing *Ca*MES_K952A_ produce superheavy ergothioneine from heavy ergothioneine and heavy TMH suggesting that sulfurization of TMH is reversible and that the MPT site is active despite an inactive desulfurase module. Right: proposed catalytic cycle: (i) base catalysis activates the imidazole ring of TMH for nucleophilic attack onto the terminal sulfido ligand at the molybdenum center; (ii) tautomerization allows ergothioneine to (iii) dissociate from the cofactor; (iv, v) the reduced metal center accepts a sulfur atom from a nearby persulfide-containing cysteine residue.

This behavior could be explained by two models: either desulfurase activity is required to supply sulfur in each turnover because the enzyme is unable to accept any other source of sulfane sulfur—such as hydrogen polysulfides or organic polysulfides—that are common cellular components.^54,55^ Alternatively, intramolecular desulfurase activity is required to keep the MPT-binding module in an active form, for example, by assisting the insertion of the Fe_4_S_4_ cluster at the N-terminus or by maintaining the proper redox state of the molybdenum center. To distinguish as to whether the desulfurase is required for turnover or for activation, we examined the functionality of the MPT-binding module in *Ca*MES_K952A_. Cells containing *Ca*MES_K952A_ were cultivated in the presence of 50 μM ^34^S-labeled heavy ergothioneine (**5**: ^34^S-ERG, *m*/*z* calcd: 232.0916; obsd: 232.0915, Figure 5) and 10 μM D_5_-^15^N_3_-labeled heavy TMH (**6**: ^2^H^15^N-TMH, *m*/*z* calcd: 206.1462; obsd: 206.1461). Quantification of the ergothioneine isotopologues revealed that these cells accumulated not only 410 ± 120 μM of ^34^S-labeled heavy ergothioneine (**5**) from the growth medium but also 55 ± 1 μM of D_4_-^15^N_3_-^34^S-labeled superheavy ergothioneine (**7**: *m*/*z* calcd: 239.1078; obsd: 239.1076, Figure S9) and only 1.7 ± 0.1 μM of ergothioneine derived from heavy TMH and ^32^S (**8**: *m*/*z* calcd: 239.1078; obsd: 239.1076, Figure S9). The latter two must have been produced by MES because they were not produced by cells containing *Ca*MES_C153S_. These observations show that cells with *Ca*MES_K952A_ can transfer ^34^S from heavy ergothioneine to heavy TMH to generate superheavy ergothioneine. Apparently, in cells containing *Ca*MES_K952A_, this shuffling process is more efficient than introducting ^32^S from alternative sulfur donors. Based on these results, we conclude that (a) the MPT-binding module in *Ca*MES_K952A_ is still active but not in *Ca*MES_C153S_ and (b) sulfurization of TMH by a sulfur-containing MPT is chemically reversible under physiological conditions (Figure 5).

## Conclusions

The discovery of MES from *C. abyssi* and other strictly anaerobic bacteria and archaea is significant for several reasons. First, this result documents the fourth case of independent emergence of ergothioneine biosynthesis. It is somewhat puzzling that the different pathways use different chemistry for C–S bond formation, whereas TMH is always produced by a methyltransferase from a single protein family (Figure 1). Since the methylation activity was available through horizontal gene transfer, why was the sulfurase reinvented? One partial explanation may be that ergothioneine biosynthesis has emerged first in an ancient anaerobic world and was then replaced by an O_2_-dependent sulfoxide synthase after the great oxygenation event made O_2_ available 2.4 billion years ago.^56^ A second explanation could be that oxidative sulfurization of heterocycles is or was a much more common microbial activity than is currently appreciated. Some of the many EanB-like enzymes,^18,57^ and mononuclear molybdenum-containing enzymes that are encoded in bacterial genomes and have no known function,^26^ may attach sulfur to heterocycles. An abundance and diversity of such enzymes with promiscuous activity for TMH may have facilitated the emergence of new ergothioneine synthases.^58^

Second, the discovery of MES in organisms from hot, reducing, and completely dark habitats—under conditions that are reminiscent of the early earth environment—adds further evidence that ergothioneine could serve cellular functions that are very different from those considered in the current literature on human physiology.^3,59−61^ Studying the metabolism of *C. abyssi* and other organisms from deep-sea hydrothermal vents may highlight new aspects of ergothioneine biochemistry. The involvement of ergothioneine as a cofactor in the biosynthesis of lincomycin A by *Streptomyces* provides an intriguing example of how diverse such functions may be.^62^

Finally, MES represents a new type of enzyme. The diverse superfamily of mononuclear molybdenum-containing enzymes has been classified into three subfamilies, represented by the enzymes xanthine oxidase, sulfite oxidase, and DMSO reductase.^23^ Known members of the third family all contain a Mo- or W-containing bis-MGD cofactor and most catalyze C–O bond formation by oxygen-atom transfer (DMSOR, EC 1.8.5.3), by hydroxylation (ethylbenzene dehydrogenase, EC 1.17.99.2),^63^ or by hydration (acetylene hydratase, EC 4.2.1.112).^32^ In addition, polysulfide reductase (EC 1.12.98.4) catalyze reductive S–S bond cleavage in the conversion of elemental sulfur (S_8_) to H_2_S and polysulfides. The C–S bond forming activity of MES is unprecedented among molybdenum-containing enzymes.^23,64^ A tentative proposal for how MES catalyzes this reaction is shown in Figure 5. The imidazole ring of TMH attacks the electrophilic sulfido ligand of the Mo^VI^ = S cofactor (i, Figure 5). Base-catalyzed tautomerization (ii) and dissociation of ergothioneine (iii) leave the enzyme in a reduced Mo^IV^ state (iv). Resulfurization by a protein-borne persulfide restores the Mo^VI^-sulfido electrophile (v). C–S bond formation with the opposite reaction polarity, namely, by attack of the sulfide from a Mo^IV^–SH onto the imidazolium ring of TMH leads to an intermediate that would require expulsion of a hydride to form ergothioneine. We consider this mechanism less likely. Irrespective of the mechanism, we anticipate that further examination of the structure and mechanism of MES will open new avenues for exploiting Mo-dependent enzymes in biocatalysis,^26^ not the least for applications in the fermentative production of ergothioneine in recombinant microorganisms.^65−68^

## Methods

### Materials

All standard reagents were purchased from Sigma–Aldrich if not otherwise stated. Expression plasmids were purchased from Biocat GmbH. Isotopologues of ergothioneine and F-TMH were produced and characterized as described in previous work.^20,47^

### Production and Purification of *Ca*MES_C-term_

The gene coding for this protein fragment was expressed from a pET28a vector. The resulting recombinant protein contained a N-terminal His_6_ tag. The plasmid was transformed into chemically competent *E. coli* BL21 pLysS (DE3) and plated onto selective agar plates. Cells were then grown in an auto-induction medium^69^ supplemented with 34 mg/L chloramphenicol and 50 mg/L kanamycin. Growing conditions: 180 rpm, 37 °C until OD_600_ reached 0.6 and then incubated at 18 °C for 24 h. Cells were then harvested by centrifugation at 4 °C at 8000 rpm for 20 min. The cells were resuspended in lysis buffer (50 mM Na_2_HPO_4_, 300 mM NaCl, pH 8.0) and lysed by sonication. The cell-free lysates were cleared by centrifugation (8000 rpm for 45 min at 4 °C). The cleared lysates were supplemented with 10 mM imidazole and incubated with a Ni-NTA resin (Qiagen GmbH) at 4 °C for 20 min. The resin was collected in a column and washed with lysis buffer supplemented with 20 mM imidazole. The His_6_-tagged protein was eluted in lysis buffer supplemented with 250 mM imidazole. Purified *Ca*MES_C-term_ was first dialyzed into Tris buffer (50 mM Tris, 200 mM NaCl, 1 mM DTT, 150 μM PLP, pH 8.0) and then into 50 mM phosphate buffer, pH 8.0. The protein was then frozen in liquid nitrogen and stored at −80 °C until use. The homogeneity of the purified protein was assessed by sodium dodecyl sulfate polyacrylamide gel electrophoresis (SDS-PAGE) (12%) analysis (Figure S3). The identity of *Ca*MES_C-term_ was confirmed by HR-ESI-MS: *m*/*z*, (calcd) 44820.4, *m*/*z* (obsd) 44820. This procedure yielded 15 mg of protein per L culture.

### Characterization of Full-Length MES in Recombinant *E. coli*

#### Transformation

Plasmids were transformed into electrocompetent *E. coli* Δmtn cells or chemically competent *E. coli* BL21 cells, spread onto selective LB-agar (50 mg/L kanamycin) plates, and incubated overnight at 37 °C. A single colony was picked, inoculated into 5 mL of LB medium, and incubated overnight at 37 °C. Overnight cultures were mixed with glycerol (total 30% glycerol) and stored at −80 °C until further use. To generate cells containing two plasmids, we first transformed electrocompetent *E. coli* Δmtn cells with the pET19b_EgtD plasmid. Individual colonies grown on selective LB-agar (100 mg/L ampicillin) plates at 37 °C were used to inoculate 5 mL of selective LB medium supplemented with 0.4% glucose. After 5 h growth at 37 °C, *ca*. 1.3 mL of culture was transferred into a sterile Eppendorf tube and centrifuged for 30 s at 12 000 rpm. The pelleted cells were washed with a cold sterile solution of 0.1 M CaCl_2_ and 10% glycerol. After centrifugation, the cells were resuspended in 100 μL of the same solution and incubated on ice for 1 h. These cells were used for transformation with the pCOLADuet-1_*Ca*MES plasmid by heat shock and further grown on agar plates supplemented with two antibiotics (100 mg/L ampicillin, 50 mg/L kanamycin).

#### Cell Cultures

Five milli-liter of preculture was grown in LB or chemically defined medium (CD, Table S1) containing the appropriate antibiotics overnight at 30 °C (180 rpm); 0.5–1 mL of preculture was used to inoculate 30–100 mL of LB or CD medium (with antibiotics). These cultures were incubated at 37 °C (180 rpm) until an optical density (OD_600_) of 0.6–0.8 was reached. IPTG was then added to a final concentration of 1 mM, and cell cultures were cooled down to 20 °C (180 rpm). From these cultures, 7 mL aliquots were transferred to sterile falcon tubes (weighted) and supplemented with Na_2_MoO_4_, Na_2_WO_4_, and/or substrates. These aliquots were grown for 20 h at 25 °C. Concentrations of supplements are listed separately for each experiment below. All additives were sterile filtered.

#### Extraction of Ergothioneine and TMH

After incubation for 20 h at 25 °C, cells were centrifuged for 30 min (2000 rpm, 4 °C), supernatant was removed, and wet cell pellets were weighed. The cells were then resuspended in 1 mL of cold methanol and extracted by sonication for 5 min in an ultrasonic bath. The crude extracts were cleared by centrifugation (30 min, 2000 rpm, 20 °C). The methanolic supernatant was dried using an Eppendorf concentrator under vacuum for 1.5 h at 60 °C. The dry residues were dissolved in milli-Q water (100 μL per 100 mg of wet pellet). Insoluble residues were removed by centrifugation. The cleared supernatant was analyzed by RP-HPLC ESI-HR-MS on a LC-coupled Bruker maXis II.

#### Quantification of Ergothioneine and TMH

To determine the concentration of ergothioneine and TMH in cell-free lysates, we used HR-ESI-MS analysis and quantified the detected signals for these compounds by comparison to signals measured for authentic standards with known concentrations. For external quantification, standard solutions of authentic ergothioneine and TMH (20 μM in H_2_O) were measured as independent samples in the same measurement series with experimental samples. This procedure allowed us to reliably quantify the concentration of analytes within an order of magnitude. However, we noticed that external calibration tends to underestimate the concentration of analytes within cellular extracts. For more precise quantification, isotopologues of desired products were used as internal standards: to quantify ergothioneine, 4 μL of supernatant was mixed with 4 μL of isotopically labeled ergothioneine of known concentration (10 or 75 μM). The ion count ratio of product/isotopologue in each sample was then calculated to obtain the concentration of the analyte. For the analysis of mass spectra, extracted ion chromatograms (EIC) (Table S2, error ± 0.001) were used on Bruker Compass HyStar software; analytes and standards were identified in the MS spectrum provided (observed *m*/*z* ± 5 ppm) (Table S3).

## Supporting Information Available

Sequences of proteins used in this study; a list of bacterial species that contain MES homologues; supporting figures (Figures S1 and S2); analytical data including NMR spectra; SDS-PAGE of purified proteins; and exemplary mass spectra (Figures S3–S9) (PDF)
