# Supplementary material for: Discovery and Characterization of the Metallopterin-Dependent Ergothioneine Synthase from Caldithrix abyssi
Source: JACS Au. 2022 Aug 16;2(9):2098–107. doi: 10.1021/jacsau.2c00365 (PMC9516567; doi:10.1021/jacsau.2c00365)
Supplement: Supplementary file 1 — au2c00365_si_001.pdf [file au2c00365_si_001.pdf]

Supplementary information for

The Discovery and Characterization of the Metallopterin-Dependent Ergothioneine Synthase from *Caldithrix abyssi*

Mariia A. Beliaeva[1,2] and Florian P. Seebeck[1,2]*

florian.seebeck@unibas.ch

[1]Department of Chemistry, University of Basel, Mattenstrasse 24a, Basel 4002, Switzerland

[2]Molecular Systems Engineering, National Competence Center in Research (NCCR), 4058 Basel, Switzerland

Sequences of proteins encoded in expression plasmids:

>*Ca*MES (*Caldithrix abyssi,* UniProt A0A7V4WV16), plasmid: pCOLADuet-1
MGHHHHHHAENLYFQGSGMSIAEPNTEKRALCGICPAGCWVKVKYDAGGRLSQVKADDSSHLGMICTLGEHSADIVYSEHRLRYPMRRKGPKGSYDFERISWDTAYD
IIVDNLQKIKKESGPEATAVYTGRGSFELAMCDVFQPKGVAVSSASSVLFPFGSPNTLGVGALCYVSFAMIAPHVTMGGMLINMFSDIERAELIVVWGANPATDSPP
LDFQRIVAAQKRGAQVVVIDPRRTKTAKVTDAQWIPVRPGTDGALALGLCNVLIEEELYDESFARDWTLGFDDFTRHVQHYRPEVVERITGVPAQTVQELARRIAAA
NGAAPVMYSGLEYSNSGVQAIRATMALWALAGQLDVPGGRCFNMRDNTFPINREGLIPNPNEAKALGRDRFPVYSAYRGESHAIALPEAVLNGDPYRIRSLIILGGS
IITAWPQPDIWRKTLKTLDFLVTIDRQLTADSAYADIVLPATTMYEIESYMTYGSMFRIREKVIEPVGEARNDFFIMAELAKRLGYGHLYPQNEDELLRYVLKGSGF
TPEEVRAKGGLVQKPTSILEYKKWEKGLLRSDGQKGFDTPSGKFELASQFLAEHGYDPLPVYTEPTEGPLSRPDLAKEFPLVFNSGSRVTTDFRSQHHGIPALNKRR
REPQVTMNSADAGERGIKSGDWVRISTRRGSVVMRAYVTDDIVAGSIDADMGGGGPVGPQAWQKCNINDVTDLQNYDPISGFPVYKTLLCQVSKVESGKDGTVLDSG
EASGTIAADNGNGRAAQPEFKRIYLDHNATTPIDPEVKKVMREYLDAGQGNPSAIYHEGKESRFAVEAARRSVARLLNCTARRIVFTGGGSEANNLAIKGIAFAHEG
KRNHIITSQIEHPAIRNTCKWLESRGFTVTYLPVDQFGRVRPHDLEKAISDRTLLVSVMLANNETGSIQPVAELARIAHQHGAYMHTDAVQAVAKIPVDVNELEVDL
LTLSGHKFYGPKGIGALYVRKDIPLTPLVHGGHQESDLRAGTENVLAMVGLGKAADLAVQRLPKMKKVEALRNHLEEEIKKIIPEARVNGHPVHRVPVTLNMTIPGM
RGESLVLALDEKGLSLSSGSACRSGSPDPSPALLAMGLSEEEAHCALRFSLGVKTTAKDIERTLHLFEEVVTDAKTMVRFIPCR

>*Ea*MES (*Euryarchaeota archaeon,* UniProt A0A1V6KBG7), plasmid: pCOLADuet-1
MGHHHHHHAENLYFQGSGMTHTVQGERRALCGICSAGCGVIVAYDDHGRIASVRPDEDAEIGIICRLGEASPEIVYSSDRVLYPLRRAGPKGTHAFERITWDEAYET
IVANLTRIREESGPEAVAIYTGSGSFELSFCDIFQPKGVAVSSASSVLFPFGSPNTMGVGALCYVSFAMIAPHVTMGEMYFNMFSDYEQSDLILVWGTNPATDCPPL
ALRALAGARRRGAEVVVIDPRRTRTVDLVDAEWVPIRPGTDGALALGLANVIVAEELYDADFVANWCHGFEEFATYVQHYRPEVVESITGIPAETVASLARRIARAR
GASFAMYTGLEYSDSGVQAIRAVFVLWGISGNLDVPGGRCFAMRGNAFPINRSGYIQNPDLKRAIGTDRFPVYTHYRQEGHAIALPDAVLRGRPYRIRALILEAAHM
LISWPQTPVWRETLANLDFLVSVDRHLTADAAYADIVLPATTLYERESYMTYGPVFRLRERVIEPLGEARGDVQIMAELARRLGYGHLYPQSEEEALRHVLKGSGFT
LEGVRAAGGTVRSPAAMMEYRKWEKGLLRPDGRPGFDTPTGKFEIYSTILEEYGYDPLPVYTEPGESPLSQPEQADRFPLVFNSGARVTTDFHAQHHSIPSLVRERP
EPTVTLNSHDARERGIRDGDRVIVRTARGEIVLRALVTDGIVRGAVEANMGGGCHQAPEAWREGNVNELTDLARYDPISGFPVYKALLCDVARAEGDGGRVAIGTGE
VDSVAGAAPPPEAPRIYLDHNATTPLDPAVHQAMAAYLTSNPGNPSSIYREGRDAKVAVESARRSLARLLNCTARRITFTGSCTEANNTVIKGLAFAGRDGIRREII
TSPTEHSAVIEPCRWLEQFGFRVTFLPVDRSGRVDPADLEGLIGPETLLVSVMVANNETGTIQPVRELVRIAHDHGALFHTDATQAIGKMPVDAVDLDVDFLTLSGH
KIYGPKGVGALYMKKGVRIAPLVHGGEQEGRFRAGTENVAGIVGLGRAAEIAEQHIGRMDAVRHLRDRLEAGIRETVPGARLNGHPTERLANTLNMVLPGYRGESIV
LALDRRGISLSSGSACHSGSPAPSHVLLAMGLSAEDAHCSIRMTLGAHTTDEDIDRTLGAFRDLLHGPGEIIRFVACR

>*Ab*MES (*Abyssubacteria bacterium*, UniProt A0A3A4NVY7), plasmid: pCOLADuet-1
MGHHHHHHAENLYFQGSGMTEISTQLAPQQDSPAEQIKDGLCGICPAGCWVRVRLNGGRMSGVKALPDHPLGMICTIGKHSPQIVHDPNRLQYPLRRTGPKGSYDFE
RITWNEAMEAIAGKLDDIKREHGPEATAIYTGRGSFELSMCDVYQPRGVAVSSASSVLFPFGSPNTLGVGALCYVSFAMIAPHVTMGEMLITMDTEIEQAELIVVWG
ANPATDSPPLAHAQILRARERGAEVVVIDPRRSETAREAESEWIPLRPGTDGALALGMINVLVEEELYDEDFAENWTLGFNELKQYVQHFRPETVEQITEVPAEKVR
SLSRRIARARGACPVMYTGLEYSDSGVQAIRAVHTLWALAGQLDVPGGLVFRMKENIFPINRSGLIANPDVRKALGRDRFPVYSMYRGESHAIALPESVLEGRPYKI
RSLIILGASIITAWPQPDLWRKTLNALDFLVCIDRYFTADAAYADIVLPATTMYEIMSYMVYGPLFIIRERIVEPVGEARNDFLILAELAKRLGYGHLYPQTEEELL
RHVLQGSGYTLEEVKAVGGEVRLPAVMMQYKKWMKGLLRPDGKPGFNTPSGKFEIASSILAEHGYDALPVYVEPGEGPLANPQLAADYPLVFNSGARVFSDFRSQHH
GVSGLREMTPDPIVTMNAVDASERGIENGDWVNVTTKRGQVKFRAIVTDDIMPGAIDACMGGGGPLGSRSWQECNVNELTDLTRYDPISGFPVYKSLLAEVVKAGVA
KSADRITESVPAKTKSRERHRPPKLRHVYLDHNATTPMDPEVVEAMMPYLRESFGNPSSIHQRGVEARDAVEGARRKVAQLINCTARRVVFTGGGSEADNLAVKGVA
FARREEGDHIVTTKIEHPAVLNSCRSLEAMGFEVTYLDVDQDGLVDPRQLEDSIRPQTTLVSIMLANNEVGTIQPIAQLARIAHERDALFHCDAVQGAGKIEVDVEK
LGVDLLSLSSHKIHGPKGMGMLYVRKGIPLVPLIDGGGQEQKLRAGTENVAGIVGFGKACELAQRKLNERDMERVQALRDALEKGLRELVPDMKLNGHPSLRLPNTL
NVTLPAMRGESLVMFLSRRGVYCSSGSACKSGNPEPSLTLLAMGLSVEDAHCALRLSLGSGNNEEDVKYMLHVLGQIIRDSRSSVRFAACR

>*hv*MES (*hydrothermal vent metagenome,* UniProt A0A3B1C0R1), plasmid: pCOLADuet-1
MGHHHHHHAENLYFQGSGMDKRTLEWKKGICGICPAGCWIEAGMQAGKMVDIRADTSHQLGMICRRGQHAPEIIYSEQRLKYPLKRVGPKGKHAFERISWDEAYDCI
VKNLNAIKAESGPEAVSIYTGRGAFELSLCDMYQPKDVAVSSASNILFPFGSPNTMGVGALCYVSFAMIAPDVTLGSMLINMYTDIENAELLFVWGANPATDSPPLD
MARLEAAAKRGCDMVVIDPRYTETAKRTGAEWIAIRPGTDGALALSMMGVLIDEDLYDEKFVEQWTHGFADLTGYVQHFRPEVVERITGVPADKIRELARRIARATG
AAPVMYTGLEYSNSGIQAVRAVLSLFAISGHLDTPGGTGLAMRGQHFPVNRSCNQQNPDLARAAARDKFPLYSDYRGESHASGLVDAVLHSEPYSIRGLIIHGASLL
TSWPQTSIWRKTLSKLDFVVSIDRQLTADAAYADIVLPATTMFEIDSYMVYGPIFRLREKLVEPVGEARNDYLIMAELAKRLGYGHLYPQTEEDMIRFALKDSGYTL
EDIQRNGGWVKLPTPMMEYKKYEKGSLRADGKPGFETPTGKFEIWSSKLEEYGYEPLPKYVEPVEGPQAAPELSQRFPLVFNSGARPQTDFRSQHHGIKGLNKDNPE
PTVEINSDDARERGIEPGDLVELRTPRGAVPFRARVSQDIVRGAIEANMGGGTPVGPKAWQEWNVNELTDLYNYDEISGFPVYKALLCEVVKIKSADAKTRLRLTDS
EKGTKADFTFKGSDRKKNRQRIYLDNNATTAIAKSVKEAMQPYLDSLFGNPSSIHSTGRDARDAVDKARRQVARLIGARPKRIIFTGGGSEANNLALKGIAFARQST
GRHIITSRVEHPAILQTTDFLERQGFRITYLEVDRRGLVQPQKLEEALTDDTILVSIMLANNEVGSIQPIRELVQIAHAQSVLFHTDAVQAAGKIPIDIEKLDVDLL
SLSGHKFHAPKGVGALYVRKGLELESLIHGGQQESGRRAGTENVAAIVGMGKAAELALHDLPKSPAMARLRDRLQNGIQALVPQSHLNGHLEQRLPNTLNMTLPGLR
GESLVVALDQHGISFSSGSACKSGSPKPTHVLMAMGCSEEDAHCAVRFSLDAGIRQKDIDVTLAELKNVLLEIETTVRFLPCK

>EgtD (*Mycolicibacterium smegmatis*, UniProt A0R5M8), plasmid: pET19b
MGSSHHHHHHSSGLVPRGSHMALSLANYLAADSAAEALRRDVRAGLTATQKSLPPKWFYDAVGSDLFDQITRLPEYYPTRTEAQILRTRSAEIISAAGADTLVELGS
GTSEKTRMLLDAMRDAELLRRFIPFDVDAGVLRSAGAAIGAEYPGIEIDAVCGDFEEHLGKIPHVGRRLVVFLGSTIGNLTPAPRAEFLSTLADTLQPGDSLLLGTD
LVKDTGRLVRAYDDAAGVTAAFNRNVLAVVNRELSADFDLDAFEHVAKWNSDEERIEMWLRARTAQHVRVAALDLEVDFAAGEEMLTEVSAKFRPENVVAELAEAGL
RQTHWWTDPAGDFGLSLAVR

>*Ca*MES$_{\text{C-term}}$ (C-terminus of *Ca*MES), plasmid: pET28a
MGHHHHHHAENLYFQGSGMAQPEFKRIYLDHNATTPIDPEVKKVMREYLDAGQGNPSAIYHEGKESRFAVEAARRSVARLLNCTARRIVFTGGGSEANNLAIKGIAF
AHEGKRNHIITSQIEHPAIRNTCKWLESRGFTVTYLPVDQFGRVRPHDLEKAISDRTLLVSVMLANNETGSIQPVAELARIAHQHGAYMHTDAVQAVAKIPVDVNEL
EVDLLTLSGHKFYGPKGIGALYVRKDIPLTPLVHGGHQESDLRAGTENVLAMVGLGKAADLAVQRLPKMKKVEALRNHLEEEIKKIIPEARVNGHPVHRVPVTLNMT
IPGMRGESLVLALDEKGLSLSSGSACRSGSPDPSPALLAMGLSEEEAHCALRFSLGVKTTAKDIERTLHLFEEVVTDAKTMVRFIPCR

>*Ca*MES$_{\text{C153S}}$, plasmid: pET28a
MGHHHHHHAENLYFQGSGMSIAEPNTEKRALCGICPAGCWVKVKYDAGGRLSQVKADDSSHLGMICTLGEHSADIVYSEHRLRYPMRRKGPKGSYDFERISWDTAYD
IIVDNLQKIKKESGPEATAVYTGRGSFELAMCDVFQPKGVAVSSASSVLFPFGSPNTLGVGALSYVSFAMIAPHVTMGGMLINMFSDIERAELIVVWGANPATDSPP
LDFQRIVAAQKRGAQVVVIDPRRTKTAKVTDAQWIPVRPGTDGALALGLCNVLIEEELYDESFARDWTLGFDDFTRHVQHYRPEVVERITGVPAQTVQELARRIAAA
NGAAPVMYSGLEYSNSGVQAIRATMALWALAGQLDVPGGRCFNMRDNTFPINREGLIPNPNEAKALGRDRFPVYSAYRGESHAIALPEAVLNGDPYRIRSLIILGGS
IITAWPQPDIWRKTLKTLDFLVTIDRQLTADSAYADIVLPATTMYEIESYMTYGSMFRIREKVIEPVGEARNDFFIMAELAKRLGYGHLYPQNEDELLRYVLKGSGF
TPEEVRAKGGLVQKPTSILEYKKWEKGLLRSDGQKGFDTPSGKFELASQFLAEHGYDPLPVYTEPTEGPLSRPDLAKEFPLVFNSGSRVTTDFRSQHHGIPALNKRR
REPQVTMNSADAGERGIKSGDWVRISTRRGSVVMRAYVTDDIVAGSIDADMGGGGPVGPQAWQKCNINDVTDLQNYDPISGFPVYKTLLCQVSKVESGKDGTVLDSG
EASGTIAADNGNGRAAQPEFKRIYLDHNATTPIDPEVKKVMREYLDAGQGNPSAIYHEGKESRFAVEAARRSVARLLNCTARRIVFTGGGSEANNLAIKGIAFAHEG
KRNHIITSQIEHPAIRNTCKWLESRGFTVTYLPVDQFGRVRPHDLEKAISDRTLLVSVMLANNETGSIQPVAELARIAHQHGAYMHTDAVQAVAKIPVDVNELEVDL
LTLSGHKFYGPKGIGALYVRKDIPLTPLVHGGHQESDLRAGTENVLAMVGLGKAADLAVQRLPKMKKVEALRNHLEEEIKKIIPEARVNGHPVHRVPVTLNMTIPGM
RGESLVLALDEKGLSLSSGSACRSGSPDPSPALLAMGLSEEEAHCALRFSLGVKTTAKDIERTLHLFEEVVTDAKTMVRFIPCR

>*Ca*MES$_{\text{K952A_a}}$, plasmid: pET28a
MGHHHHHHAENLYFQGSGMSIAEPNTEKRALCGICPAGCWVKVKYDAGGRLSQVKADDSSHLGMICTLGEHSADIVYSEHRLRYPMRRKGPKGSYDFERISWDTAYD
IIVDNLQKIKKESGPEATAVYTGRGSFELAMCDVFQPKGVAVSSASSVLFPFGSPNTLGVGALCYVSFAMIAPHVTMGGMLINMFSDIERAELIVVWGANPATDSPP
LDFQRIVAAQKRGAQVVVIDPRRTKTAKVTDAQWIPVRPGTDGALALGLCNVLIEEELYDESFARDWTLGFDDFTRHVQHYRPEVVERITGVPAQTVQELARRIAAA
NGAAPVMYSGLEYSNSGVQAIRATMALWALAGQLDVPGGRCFNMRDNTFPINREGLIPNPNEAKALGRDRFPVYSAYRGESHAIALPEAVLNGDPYRIRSLIILGGS
IITAWPQPDIWRKTLKTLDFLVTIDRQLTADSAYADIVLPATTMYEIESYMTYGSMFRIREKVIEPVGEARNDFFIMAELAKRLGYGHLYPQNEDELLRYVLKGSGF
TPEEVRAKGGLVQKPTSILEYKKWEKGLLRSDGQKGFDTPSGKFELASQFLAEHGYDPLPVYTEPTEGPLSRPDLAKEFPLVFNSGSRVTTDFRSQHHGIPALNKRR
REPQVTMNSADAGERGIKSGDWVRISTRRGSVVMRAYVTDDIVAGSIDADMGGGGPVGPQAWQKCNINDVTDLQNYDPISGFPVYKTLLCQVSKVESGKDGTVLDSG
EASGTIAADNGNGRAAQPEFKRIYLDHNATTPIDPEVKKVMREYLDAGQGNPSAIYHEGKESRFAVEAARRSVARLLNCTARRIVFTGGGSEANNLAIKGIAFAHEG
KRNHIITSQIEHPAIRNTCKWLESRGFTVTYLPVDQFGRVRPHDLEKAISDRTLLVSVMLANNETGSIQPVAELARIAHQHGAYMHTDAVQAVAKIPVDVNELEVDL

LTLSGHAFYGPKGIGALYVRKDIPLTPLVHGGHQESDLRAGTENVLAMVGLGKAADLAVQRLPKMKKVEALRNHLEEEIKKIIPEARVNGHPVHRVPVTLNMTIPGM
RGESLVLALDEKGLSLSSGSACRSGSPDPSPALLAMGLSEEEAHCALRFSLGVKTTAKDIERTLHLFEEVVTDAKTMVRFIPCR

>*Ca*MES$_{K952A_b}$, plasmid: pETDuet-1
MGHHHHHHAENLYFQGSGMSIAEPNTEKRALCGICPAGCWVKVKYDAGGRLSQVKADDSSHLGMICTLGEHSADIVYSEHRLRYPMRRKGPKGSYDFERISWDTAYD
IIVDNLQKIKKESGPEATAVYTGRGSFELAMCDVFQPKGVAVSSASSVLFPFGSPNTLGVGALCYVSFAMIAPHVTMGGMLINMFSDIERAELIVVWGANPATDSPP
LDFQRIVAAQKRGAQVVVIDPRRTKTAKVTDAQWIPVRPGTDGALALGLCNVLIEEELYDESFARDWTLGFDDFTRHVQHYRPEVVERITGVPAQTVQELARRIAAA
NGAAPVMYSGLEYSNSGVQAIRATMALWALAGQLDVPGGRCFNMRDNTFPINREGLIPNPNEAKALGRDRFPVYSAYRGESHAIALPEAVLNGDPYRIRSLIILGGS
IITAWPQPDIWRKTLKTLDFLVTIDRQLTADSAYADIVLPATTMYEIESYMTYGSMFRIREKVIEPVGEARNDFFIMAELAKRLGYGHLYPQNEDELLRYVLKGSGF
TPEEVRAKGGLVQKPTSILEYKKWEKGLLRSDGQKGFDTPSGKFELASQFLAEHGYDPLPVYTEPTEGPLSRPDLAKEFPLVFNSGSRVTTDFRSQHHGIPALNKRR
REPQVTMNSADAGERGIKSGDWVRISTRRGSVVMRAYVTDDIVAGSIDADMGGGGPVGPQAWQKCNINDVTDLQNYDPISGFPVYKTLLCQVSKVESGKDGTVLDSG
EASGTIAADNGNGRAAQPEFKRIYLDHNATTPIDPEVKKVMREYLDAGQGNPSAIYHEGKESRFAVEAARRSVARLLNCTARRIVFTGGGSEANNLAIKGIAFAHEG
KRNHIITSQIEHPAIRNTCKWLESRGFTVTYLPVDQFGRVRPHDLEKAISDRTLLVSVMLANNETGSIQPVAELARIAHQHGAYMHTDAVQAVAKIPVDVNELEVDL
LTLSGHAFYGPKGIGALYVRKDIPLTPLVHGGHQESDLRAGTENVLAMVGLGKAADLAVQRLPKMKKVEALRNHLEEEIKKIIPEARVNGHPVHRVPVTLNMTIPGM
RGESLVLALDEKGLSLSSGSACRSGSPDPSPALLAMGLSEEEAHCALRFSLGVKTTAKDIERTLHLFEEVVTDAKTMVRFIPCR

>*Ca*MES$_{C1074S}$, plasmid: pET28a
MGHHHHHHAENLYFQGSGMSIAEPNTEKRALCGICPAGCWVKVKYDAGGRLSQVKADDSSHLGMICTLGEHSADIVYSEHRLRYPMRRKGPKGSYDFERISWDTAYD
IIVDNLQKIKKESGPEATAVYTGRGSFELAMCDVFQPKGVAVSSASSVLFPFGSPNTLGVGALCYVSFAMIAPHVTMGGMLINMFSDIERAELIVVWGANPATDSPP
LDFQRIVAAQKRGAQVVVIDPRRTKTAKVTDAQWIPVRPGTDGALALGLCNVLIEEELYDESFARDWTLGFDDFTRHVQHYRPEVVERITGVPAQTVQELARRIAAA
NGAAPVMYSGLEYSNSGVQAIRATMALWALAGQLDVPGGRCFNMRDNTFPINREGLIPNPNEAKALGRDRFPVYSAYRGESHAIALPEAVLNGDPYRIRSLIILGGS
IITAWPQPDIWRKTLKTLDFLVTIDRQLTADSAYADIVLPATTMYEIESYMTYGSMFRIREKVIEPVGEARNDFFIMAELAKRLGYGHLYPQNEDELLRYVLKGSGF
TPEEVRAKGGLVQKPTSILEYKKWEKGLLRSDGQKGFDTPSGKFELASQFLAEHGYDPLPVYTEPTEGPLSRPDLAKEFPLVFNSGSRVTTDFRSQHHGIPALNKRR
REPQVTMNSADAGERGIKSGDWVRISTRRGSVVMRAYVTDDIVAGSIDADMGGGGPVGPQAWQKCNINDVTDLQNYDPISGFPVYKTLLCQVSKVESGKDGTVLDSG
EASGTIAADNGNGRAAQPEFKRIYLDHNATTPIDPEVKKVMREYLDAGQGNPSAIYHEGKESRFAVEAARRSVARLLNCTARRIVFTGGGSEANNLAIKGIAFAHEG
KRNHIITSQIEHPAIRNTCKWLESRGFTVTYLPVDQFGRVRPHDLEKAISDRTLLVSVMLANNETGSIQPVAELARIAHQHGAYMHTDAVQAVAKIPVDVNELEVDL
LTLSGHKFYGPKGIGALYVRKDIPLTPLVHGGHQESDLRAGTENVLAMVGLGKAADLAVQRLPKMKKVEALRNHLEEEIKKIIPEARVNGHPVHRVPVTLNMTIPGM
RGESLVLALDEKGLSLSSGSASRSGSPDPSPALLAMGLSEEEAHCALRFSLGVKTTAKDIERTLHLFEEVVTDAKTMVRFIPCR

>*Ca*MES$_{C1097S}$, plasmid: pET28a
MGHHHHHHAENLYFQGSGMSIAEPNTEKRALCGICPAGCWVKVKYDAGGRLSQVKADDSSHLGMICTLGEHSADIVYSEHRLRYPMRRKGPKGSYDFERISWDTAYD
IIVDNLQKIKKESGPEATAVYTGRGSFELAMCDVFQPKGVAVSSASSVLFPFGSPNTLGVGALCYVSFAMIAPHVTMGGMLINMFSDIERAELIVVWGANPATDSPP
LDFQRIVAAQKRGAQVVVIDPRRTKTAKVTDAQWIPVRPGTDGALALGLCNVLIEEELYDESFARDWTLGFDDFTRHVQHYRPEVVERITGVPAQTVQELARRIAAA
NGAAPVMYSGLEYSNSGVQAIRATMALWALAGQLDVPGGRCFNMRDNTFPINREGLIPNPNEAKALGRDRFPVYSAYRGESHAIALPEAVLNGDPYRIRSLIILGGS
IITAWPQPDIWRKTLKTLDFLVTIDRQLTADSAYADIVLPATTMYEIESYMTYGSMFRIREKVIEPVGEARNDFFIMAELAKRLGYGHLYPQNEDELLRYVLKGSGF
TPEEVRAKGGLVQKPTSILEYKKWEKGLLRSDGQKGFDTPSGKFELASQFLAEHGYDPLPVYTEPTEGPLSRPDLAKEFPLVFNSGSRVTTDFRSQHHGIPALNKRR
REPQVTMNSADAGERGIKSGDWVRISTRRGSVVMRAYVTDDIVAGSIDADMGGGGPVGPQAWQKCNINDVTDLQNYDPISGFPVYKTLLCQVSKVESGKDGTVLDSG
EASGTIAADNGNGRAAQPEFKRIYLDHNATTPIDPEVKKVMREYLDAGQGNPSAIYHEGKESRFAVEAARRSVARLLNCTARRIVFTGGGSEANNLAIKGIAFAHEG
KRNHIITSQIEHPAIRNTCKWLESRGFTVTYLPVDQFGRVRPHDLEKAISDRTLLVSVMLANNETGSIQPVAELARIAHQHGAYMHTDAVQAVAKIPVDVNELEVDL
LTLSGHKFYGPKGIGALYVRKDIPLTPLVHGGHQESDLRAGTENVLAMVGLGKAADLAVQRLPKMKKVEALRNHLEEEIKKIIPEARVNGHPVHRVPVTLNMTIPGM
RGESLVLALDEKGLSLSSGSACRSGSPDPSPALLAMGLSEEEAHSALRFSLGVKTTAKDIERTLHLFEEVVTDAKTMVRFIPCR

>*Ca*MES$_{C1135S}$, plasmid: pET28a
MGHHHHHHAENLYFQGSGMSIAEPNTEKRALCGICPAGCWVKVKYDAGGRLSQVKADDSSHLGMICTLGEHSADIVYSEHRLRYPMRRKGPKGSYDFERISWDTAYD
IIVDNLQKIKKESGPEATAVYTGRGSFELAMCDVFQPKGVAVSSASSVLFPFGSPNTLGVGALCYVSFAMIAPHVTMGGMLINMFSDIERAELIVVWGANPATDSPP
LDFQRIVAAQKRGAQVVVIDPRRTKTAKVTDAQWIPVRPGTDGALALGLCNVLIEEELYDESFARDWTLGFDDFTRHVQHYRPEVVERITGVPAQTVQELARRIAAA
NGAAPVMYSGLEYSNSGVQAIRATMALWALAGQLDVPGGRCFNMRDNTFPINREGLIPNPNEAKALGRDRFPVYSAYRGESHAIALPEAVLNGDPYRIRSLIILGGS
IITAWPQPDIWRKTLKTLDFLVTIDRQLTADSAYADIVLPATTMYEIESYMTYGSMFRIREKVIEPVGEARNDFFIMAELAKRLGYGHLYPQNEDELLRYVLKGSGF
TPEEVRAKGGLVQKPTSILEYKKWEKGLLRSDGQKGFDTPSGKFELASQFLAEHGYDPLPVYTEPTEGPLSRPDLAKEFPLVFNSGSRVTTDFRSQHHGIPALNKRR
REPQVTMNSADAGERGIKSGDWVRISTRRGSVVMRAYVTDDIVAGSIDADMGGGGPVGPQAWQKCNINDVTDLQNYDPISGFPVYKTLLCQVSKVESGKDGTVLDSG
EASGTIAADNGNGRAAQPEFKRIYLDHNATTPIDPEVKKVMREYLDAGQGNPSAIYHEGKESRFAVEAARRSVARLLNCTARRIVFTGGGSEANNLAIKGIAFAHEG
KRNHIITSQIEHPAIRNTCKWLESRGFTVTYLPVDQFGRVRPHDLEKAISDRTLLVSVMLANNETGSIQPVAELARIAHQHGAYMHTDAVQAVAKIPVDVNELEVDL
LTLSGHKFYGPKGIGALYVRKDIPLTPLVHGGHQESDLRAGTENVLAMVGLGKAADLAVQRLPKMKKVEALRNHLEEEIKKIIPEARVNGHPVHRVPVTLNMTIPGM
RGESLVLALDEKGLSLSSGSACRSGSPDPSPALLAMGLSEEEAHCALRFSLGVKTTAKDIERTLHLFEEVVTDAKTMVRFIPSR

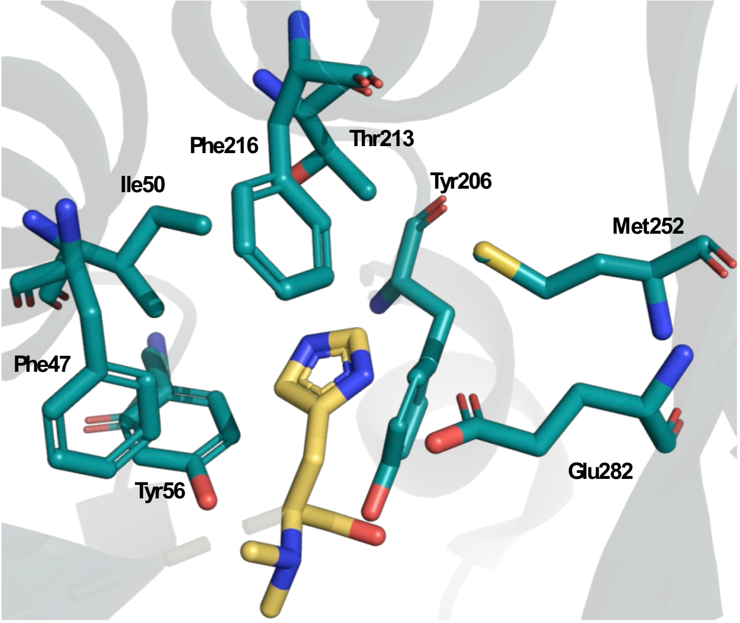


```
A0A3A4NKW8      MRSQADPSKINELPACVAIALNGGDSAIIDAAEEAN-----PVLDFAHSAARTLSENPKW
4PIO            ----------------GHMALSLANYLAADSAAEAL----------RRDVRAGLTATQKS
A0A7V4UF19      ----------------MSTNLSIADTAILERFEEILLARPVHKNAFITDVQNGLSASPKT
                                    *. .:    :   *              ..   *: . *

A0A3A4NKW8      MECRFLYDARGSALFEQICRQPEYYPTRRETAILHRYAGEICETTGCVT-LAELGCGSAI
4PIO            LPPKWFYDAVGSDLFDQITRLPEYYPTRTEAQILRTRSAEIISAAGADT-LVELGSGTSE
A0A7V4UF19      LPCIYFYDERGSQLFEDICRLPEYYLTRCEAEILRQHSREIAASFPADTLLVELGSGSSV
                :   ::**  ** **::* * **** ** *: **.  : **  :  . * *.***.*::

A0A3A4NKW8      KTRHILSAYVKSNGSVRYVPVDVSRSALQHACAAITAWHPAVKVAGIRGTYECAFPFLKA
4PIO            KTRMLLDAMRDAELLRRFIPFDVDAGVLRSAGAAIGAEYPGIEIDAVCGDFEEHLGKIPH
A0A7V4UF19      KTRYLLEAFMERHGQALYNPIDISHSILRQSALELLAHYPELKITAVAATYQEGLRRITD
                *** :*.*  .      : *.*:. .*. :   : * :* ::: .: . ::  :  :

A0A3A4NKW8      LSP--VMVLFLGSTIGNLNEDQDRLFWRNIGRHLSEGDFFLLGVDLVKDTSILEAAYNDR
4PIO            VGR--RLVVFLGSTIGNLTPAPRAEFLSTLADTLQPGDSLLLGTDLVKDTGRLVRAYDDA
A0A7V4UF19      IPARTRLIMWLGSSIGNLGRQEAVEFLGQLRGIMKPQDHLLIGIDLKKDINILKPAYDDA
                :     ::::***:****       *   :   :.  * :*:* ** ** . *  **:*

A0A3A4NKW8      AGVSAAFTLNLFERMNRELKTGIDVLHLQHVAYFSSAKSRIEIFAQFNREQRVRIKPLNK
4PIO            AGVTAAFNRNVLAVVNRELSADFDLDAFEHVAKWNSDEERIEMWLRARTAQHVRVAALDL
A0A7V4UF19      QGVTAAFNLNVLERMNRELVADFDLNAFAHLCRYNEEQGRIEMHILSLKDQIVRFRQPAF
                 **:***. *::  :**** :.:*:  : *:. :.. : ***:       * **.

A0A3A4NKW8      SIPISAGERIQVEISRKFNLNELVPRLYSCGFATRRIFSDENSWFALLLLEKVAND
4PIO            EVDFAAGEEMLTEVSCKFRPENVVAELAEAGLRQTHWWTDPAGDFGLSLA--VR--
A0A7V4UF19      RVAFDQGETIHTENSHKYAPAQIQTLARRSGLRIVKQWFDSRKWFSLNLL--AASE
                 : :  ** : .* * *:   :: .    .*:   . : *    *.* *   .
```

Figure S1. Top*:* the crystal structure of EgtD from *M. smegmatis* in complex with the reaction intermediate *Nα,Nα*-dimethylhistidine (yellow, PDB: 4PIO)[1]. Residues in direct contact with the substrate are shown in stick representation (teal). **Bottom***:* Sequence alignment of EgtD from *M. smegmatis* and the homologous methyltransferase from *Candidatus Abyssubacteria bacterium* (UniProt A0A3A4NKW8) and and *Caldithrix abyssi* (A0A7V4UF19). Most active site residues are conserved (green), or very similar.

Table S1. List of species with a gene that encodes MES homologs with the catalytic residues Cys153, Lys952, Cys1074, Cys1097 and Cys1135 conserved (*Ca*MES numbering).

UniProt ID	Organism	Phylum
A0A399XDS8	*Acidobacteria bacterium*	Acidobacteria
A0A6A0IP45	*Acidobacteria bacterium*	Acidobacteria
A0A6G0A229	*Actinobacteria bacterium*	Actinobacteria
A0A523V0G2	*Actinobacteria bacterium*	Actinobacteria
A0A3M1PJS3	*Calditrichaeota bacterium*	Calditrichaeota
A0A3M2FG68	*Calditrichaeota bacterium*	Calditrichaeota
A0A6H9L4H4	*Calditrichaeota bacterium*	Calditrichaeota
A0A7V4WV16	*Caldithrix abyssi*	Calditrichaeota
A0A3A4NVY7	*Candidatus Abyssubacteria bacterium SURF_5*	Candidatus Abyssubacteria
A0A524AS98	*Dehalococcoidia bacterium*	Chloroflexi
A0A523UH11	*Dehalococcoidia bacterium*	Chloroflexi
A0A2D6J0S4	*Dehalococcoidales bacterium*	Chloroflexi
A0A523SK89	*Dehalococcoidia bacterium*	Chloroflexi
A0A7J4FR07	*Methanosarcinales archaeon*	Euryarchaeota
A0A7K4BI03	*Methanomicrobiales archaeon*	Euryarchaeota
A0A7K4DD47	*Methanomicrobiales archaeon*	Euryarchaeota
A0A1V6KBG7	*Euryarchaeota archaeon ADurb.Bin009*	Euryarchaeota
A0A843KNK4	*Methanoculleus sp.*	Euryarchaeota
A0A498H2I6	*Methanoculleus taiwanensis*	Euryarchaeota
A0A2D5ZQU6	*Gemmatimonadetes bacterium*	Gemmatimonadetes
A0A0S8G1P3	*Gemmatimonas sp. SG8_38_2*	Gemmatimonadetes
A0A0S8HDI0	*Gemmatimonas sp. SM23_52*	Gemmatimonadetes
A0A7V1AJK2	*Nitrospirae bacterium*	Nitrospirae
A0A1G1GVX5	*Nitrospirae bacterium GWC2_57_9*	Nitrospirae
A0A523NF38	*Planctomycetes bacterium*	Planctomycetes
I3IKG5	*Candidatus Jettenia caeni*	Planctomycetes
A0A6L3AHQ7	*Candidatus Jettenia sp. AMX1*	Planctomycetes
A0A533Q600	*Candidatus Jettenia ecosi*	Planctomycetes
A0A5C6DKM7	*Candidatus Brocadiaceae bacterium S225*	Planctomycetes
A0A2C9CAM6	*Kuenenia stuttgartiensis*	Planctomycetes
A0A6G7GT36	*Kuenenia stuttgartiensis*	Planctomycetes
A0A0B0EG58	*Candidatus Scalindua brodae*	Planctomycetes
A0A286U2N3	*Candidatus Scalindua japonica*	Planctomycetes
Q1Q5D6	*Kuenenia stuttgartiensis*	Planctomycetes
A0A523CMZ8	*Candidatus Scalindua sp. AMX11*	Planctomycetes
A0A7Y3ULB5	*Planctomycetes bacterium*	Planctomycetes
A0A3A4XW55	*Desulfobacteraceae bacterium*	Proteobacteria
A0A7C1TE37	*Gammaproteobacteria bacterium*	Proteobacteria
A0A2A5HFC2	*Alteromonadaceae bacterium*	Proteobacteria
A0A1W7QPD6	*Geobacter pelophilus*	Proteobacteria
A0A6F9X0J6	*Geobacter sp. SVR*	Proteobacteria
C6E0P4	*Geobacter sp. (strain M21)*	Proteobacteria

A0A523K4L2	*Gammaproteobacteria bacterium*	Proteobacteria
A0A450YCV5	*Candidatus Kentron sp. SD*	Proteobacteria
A0A450YII0	*Candidatus Kentron sp. SD*	Proteobacteria
A0A451BQ29	*Candidatus Kentron sp. SD*	Proteobacteria
B5E8L6	*Geobacter bemidjiensis (strain Bem / ATCC BAA-1014 / DSM 16622)*	Proteobacteria
A0A257SS02	*Chromatiales bacterium 21-64-14*	Proteobacteria
B9M6I9	*Geobacter daltonii (strain DSM 22248 / JCM 15807 / FRC-32)*	Proteobacteria
I4C6F5	*Desulfomonile tiedjei (strain ATCC 49306 / DSM 6799 / DCB-1)*	Proteobacteria
A0A450U8Z3	*Candidatus Kentron sp. LFY*	Proteobacteria
A0A450U507	*Candidatus Kentron sp. LFY*	Proteobacteria
A0A450W825	*Candidatus Kentron sp. LFY*	Proteobacteria
A0A1F3Y2H8	*Bdellovibrionales bacterium RIFOXYD1_FULL_55_31*	Proteobacteria
A0A3C0YC61	*Polaromonas sp.*	Proteobacteria
A0A6P1DUA6	*Thiorhodococcus mannitoliphagus*	Proteobacteria
L0GVW7	*Thioflavicoccus mobilis 8321*	Proteobacteria
A0L7Z7	*Magnetococcus marinus (strain ATCC BAA-1437 / JCM 17883 / MC-1)*	Proteobacteria
A0A6V8MUX4	*Geomonas paludis*	Proteobacteria
A0A7C1TU51	*Gammaproteobacteria bacterium*	Proteobacteria
A0A3A5AAI7	*Desulfobacteraceae bacterium*	Proteobacteria
A0A2N2H090	*Deltaproteobacteria bacterium HGW-Deltaproteobacteria-21*	Proteobacteria
A0A0F2R6L8	*Desulfatitalea sp. BRH_c12*	Proteobacteria
A0A2N2KCG5	*Deltaproteobacteria bacterium HGW-Deltaproteobacteria-15*	Proteobacteria
A0A451AXR2	*Candidatus Kentron sp. UNK*	Proteobacteria
A0A3D3G9C8	*Bdellovibrionales bacterium*	Proteobacteria
A0A450WRK2	*Candidatus Kentron sp. LPFa*	Proteobacteria
A0A0K9I9K1	*Desulfocarbo indianensis*	Proteobacteria
A0A212PG69	*Geobacter sp. DSM 9736*	Proteobacteria
A0A1V5H0L3	*Deltaproteobacteria bacterium ADurb.BinA179*	Proteobacteria
A0A1F3T9T9	*Bdellovibrionales bacterium GWB1_55_8*	Proteobacteria
A0A6M0JW57	*Thiorhodococcus minor*	Proteobacteria
A0A1S7LH69	*Magnetococcus massalia (strain MO-1)*	Proteobacteria
A0A0S8CVX0	*Desulfobacterales bacterium SG8_35_2*	Proteobacteria
A0A2N6C8B1	*Desulfobulbaceae bacterium*	Proteobacteria
A0A1G5QK56	*Thiohalomonas denitrificans*	Proteobacteria
A0A2H6K442	*bacterium BMS3Abin01*	
A0A2H6JEA0	*bacterium BMS3Bbin12*	
A0A3B1C0R1	*hydrothermal vent metagenome*	
A0A2H6F6M9	*bacterium BMS3Abin06*	
A0A2H6ED39	*bacterium BMS3Abin03*	
A0A485M3T7	*anaerobic digester metagenome*	
A0A3B0YYM1	*hydrothermal vent metagenome*	
A0A3B0Z7Q1	*hydrothermal vent metagenome*	

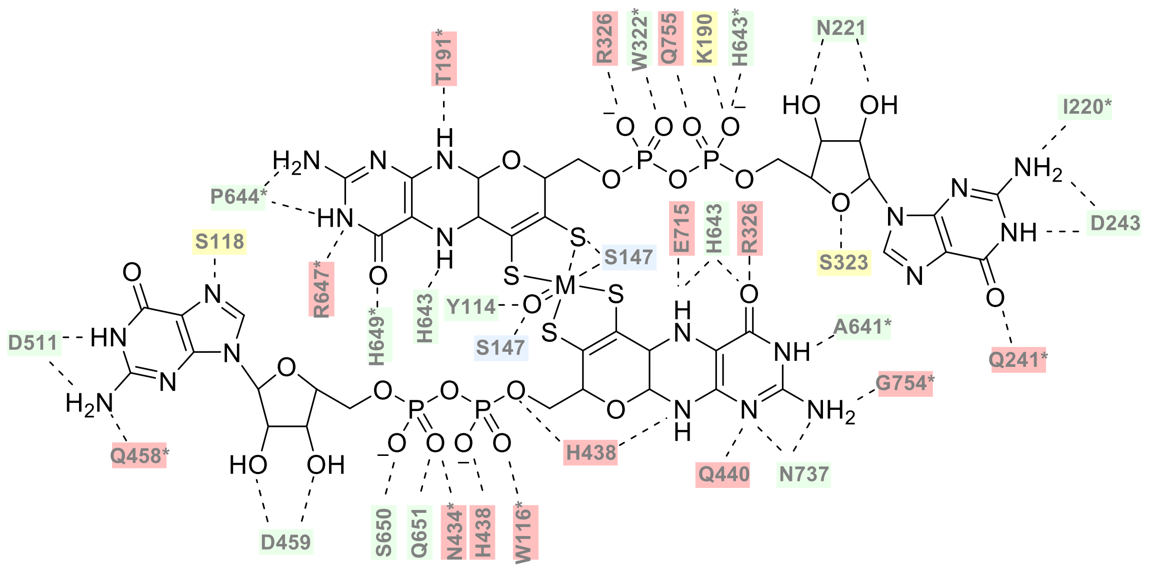

* - interaction with the peptide backbone atoms

```
DMSOreductase           MTKLSGQELHAELSRRAFLSYTAAVGALGLCGTSLLAQGARAEGLANGEVMSGCHWGVFK  60
AcetyleneHydratase      ----------------------MASKKHVVCQSCDI----------------NCVV---E  19
hvMES                   ------------------MDKRTLEWKKGICGICPA----------------GCWI---E  23
EaMES                   ------------------MTHTVQGERRALCGICSA----------------GCGV---I  23
CaMES                   -----------------MSIAEPNTEKRALCGICPA----------------GCWV---K  24
AbMES                   MTEISTQLAP-------QQDSPAEQIKDGLCGICPA----------------GCWV---R  34
                                                     :*  .                  .*

DMSOreductase           ARVE-NGRAVAFEPWDKDPAPSHQLP-------GVLDSIYSPTRIKYPMVRREFLEKGVN  112
AcetyleneHydratase      AEVKADGKIQTKSI--SEPHPTTPPNSICMKSVNADTIRTHKDRVLYPLKNV--------  69
hvMES                   AGMQ-AGKMVDIR-----ADTSHQLGMICRRGQHAPEIIYSEQRLKYPLKRV--------  69
EaMES                   VAYDDHGRIASVR-----PDEDAEIGIICRLGEASPEIVYSSDRVLYPLRRA--------  70
CaMES                   VKYDAGGRLSQVK-----ADDSSHLGMICTLGEHSADIVYSEHRLRYPMRRK--------  71
AbMES                   VRLN-GGRMSGVK-----ALPDHPLGMICTIGKHSPQIVHDPNRLQYPLRRT--------  80
                        .  .  *:                                   *: **: .

                                                          114 118
DMSOreductase           ADRSTRGNGDFVRVTWDEALDLVARELKRVQESYGPTGTFGGSYGWKSPGRLHNCQVLMR  172
AcetyleneHydratase      --GSKRGEQRWERISWDQALDEIAEKLKKIIAKYGPESLG--VSQTE---------INQQ  116
hvMES                   --GPK-GKHAFERISWDEAYDCIVKNLNAIKAESGPEAVS--IYTGRGAFELSLCDMYQP  124
EaMES                   --GPK-GTHAFERITWDEAYETIVANLTRIREESGPEAVA--IYTGSGSFELSFCDIFQP  125
CaMES                   --GPK-GSYDFERISWDTAYDIIVDNLQKIKKESGPEATA--VYTGRGSFELAMCDVFQP  126
AbMES                   --GPK-GSYDFERITWNEAMEAIAGKLDDIKREHGPEATA--IYTGRGSFELSMCDVYQP  135
                            . *   : *::*: * : :. :*  :  . ** .                  :

                                                 147
DMSOreductase           RALN--LAGGFVNSSGDY--------STAAAQIIMPHVMGTLEVYEQQTAWPVVVENTDL  222
AcetyleneHydratase      SE--YGTLRRFMNLLGSPNWTSAMYMCIGNTAG-VHRVTHGSY----SFASF---ADSNC  166
hvMES                   KDVAVSSASNILFPFGSPNTMGVGALCYVSFAMIAPDVTLGSM----LINMYTDIENAEL  180
EaMES                   KGVAVSSASSVLFPFGSPNTMGVGALCYVSFAMIAPHVTMGEM----YFNMFSDYEQSDL  181
CaMES                   KGVAVSSASSVLFPFGSPNTLGVGALCYVSFAMIAPHVTMGGM----LINMFSDIERAEL  182
AbMES                   RGVAVSSASSVLFPFGSPNTLGVGALCYVSFAMIAPHVTMGEM----LITMDTEIEQAEL  191
                                  .:   *.         .          *                   ::

                                190                           220
DMSOreductase           MVFWAADPMKTNEIGWVIPDHGAYAGMKALKEKGTRVICINPVRTETADYFGADVVSPRP  282
AcetyleneHydratase      LLFIGKNLSNHNWV-------SQFNDLKAALKRGCKLIVIDPRRTKVAEMA-DIWLPLRY  218
hvMES                   LFVWGANPATDSPP-------LDMARLEAAAKRGCDMVVIDPRYTETAKRTGAEWIAIRP  233
EaMES                   ILVWGTNPATDCPP-------LALRALAGARRRGAEVVVIDPRRTRTVDLVDAEWVPIRP  234
CaMES                   IVVWGANPATDSPP-------LDFQRIVAAQKRGAQVVVIDPRRTKTAKVTDAQWIPVRP  235
AbMES                   IVVWGANPATDSPP-------LAHAQILRARERGAEVVVIDPRRSETAREAESEWIPLRP  244
                        :.. . :  .                :    .:*  :: ::*  :...       :  *

                       241
DMSOreductase           QTDVALMLGMAHTLYSEDLHDKDFLENCTTGFDLFAAYLTGESDGTPKTAEWAAEICGLP  342
AcetyleneHydratase      GTDAALFLGMINVIINEQLYDKEFVENWCVGFEELKERVQEY------PLDKVAEITGCD  272
hvMES                   GTDGALALSMMGVLIDEDLYDEKFVEQWTHGFADLTGYVQHF------RPEVVERITGVP  287
EaMES                   GTDGALALGLANVIVAEELYDADFVANWCHGFEEFATYVQHY------RPEVVESITGIP  288
CaMES                   GTDGALALGLCNVLIEEELYDESFARDWTLGFDDFTRHVQHY------RPEVVERITGVP  289
AbMES                   GTDGALALGMINVLVEEELYDEDFAENWTLGFNELKQYVQHF------RPETVEQITEVP  298
                         ** ** *.:  .:   *:*:* .*  :   **  :   :           : .  *

                                            322  326
DMSOreductase           AEQIRELARSFVAGRTMLAAGW-SIQRMHHGEQAHWMLVTLASMIGQIGLPGGGFGLSYH  401
AcetyleneHydratase      AGEIRKAAVMFATESPASIPWAVSTDMQKNSCSAIRAQCILRAIVGSFVNGAEILGAPHS  332
hvMES                   ADKIRELARRIARATGAAPVMYTGLEYSNSGIQAVRAVLSLFAISGHLDTPGGTGLAMRG  347
EaMES                   AETVASLARRIARARGASFAMYTGLEYSDSGVQAIRAVFVLWGISGNLDVPGGRCFAMRG  348
```

```
CaMES                   AQTVQELARRIAAANGAAPVMYSGLEYSNSGVQAIRATMALWALAGQLDVPGGRCFNMRD  349
AbMES                   AEKVRSLSRRIARARGACPVMYTGLEYSDSGVQAIRAVHTLWALAGQLDVPGGLVFRMKE  358
                        *  : . :  :.           . :  . . .*      * .: * :   .

DMSOreductase           YSNGGSPTSDGP------------ALG--------------GISDGGKAVEGAAWLSESG  435
AcetyleneHydratase      ---DLVPISKIQMHEALPEEKKKLQLGTETYPFLT-YTGMSALEEPSERVYGVKYFHNMG  388
hvMES                   ---QHFPVNRSC---NQQNPDLARAAARDKFPLYSDYRGE--------------------  381
EaMES                   ---NAFPINRSG---YIQNPDLKRAIGTDRFPVYTHYRQE--------------------  382
CaMES                   ---NTFPINREG---LIPNPNEAKALGRDRFPVYSAYRGE--------------------  383
AbMES                   ---NIFPINRSG---LIANPDVRKALGRDRFPVYSMYRGE--------------------  392
                              * .                 .
                                                       438
DMSOreductase           ATSIPCARVVDMLLNPGGEFQFNGATATYPDVKLAYWAGGNPFAHHQDRNRMLKAWEKLE  495
AcetyleneHydratase      AFM----------ANPTALFTAMATEKPYPV-KAFFALASNALMGYANQQNALKGLMNQD  437
hvMES                   -------------SHASGLVDAVLHSEPYSI-RGLIIHGASLLTSWPQTSIWRKTLSKLD  427
EaMES                   -------------GHAIALPDAVLRGRPYRI-RALILEAAHMLISWPQTPVWRETLANLD  428
CaMES                   -------------SHAIALPEAVLNGDPYRI-RSLIILGGSIITAWPQPDIWRKTLKTLD  429
AbMES                   -------------SHAIALPESVLEGRPYKI-RSLIILGASIITAWPQPDLWRKTLNALD  438
                                      :  .          *   :     ..  :    :     :     :
                           458                                                  511
DMSOreductase           TFIVQDFQWTATARHADIVLPATTSYERNDIESVGDYSNRAILAMKKVVDPLYEARSDYD  555
AcetyleneHydratase      LVVCYDQFMTPTAQLADYVLPGDHWLERPVVQPNWEGIPF-GNTSQQVVEPAGEAKDEYY  496
hvMES                   FVVSIDRQLTADAAYADIVLPATTMFEIDSYMV---YGPI-FRLREKLVEPVGEARNDYL  483
EaMES                   FLVSVDRHLTADAAYADIVLPATTLYERESYMT---YGPV-FRLRERVIEPLGEARGDVQ  484
CaMES                   FLVTIDRQLTADSAYADIVLPATTMYEIESYMT---YGSM-FRIREKVIEPVGEARNDFF  485
AbMES                   FLVCIDRYFTADAAYADIVLPATTMYEIMSYMV---YGPL-FIIRERIVEPVGEARNDFL  494
                         .:  *   *  :  ** ***.    *                  :::::*  **:.:

DMSOreductase           IFAALAERLGKGAEFTEGRD-EM-GWI--SSFYEAAVKQAEFKNVAMPSFEDFWSEGIVE  611
AcetyleneHydratase      FIRELAVRMGLEEHFPWKDRLELINYRISPTGMEWEEYQKQY--------------TY--  540
hvMES                   IMAELAKRLGYGHLYPQTEE-DMIRFALKDSGYTLEDIQRNG--------------GWVK  528
EaMES                   IMAELARRLGYGHLYPQSEE-EALRHVLKGSGFTLEGVRAAG--------------GTVR  529
CaMES                   IMAELAKRLGYGHLYPQNED-ELLRYVLKGSGFTPEEVRAKG--------------GLVQ  530
AbMES                   ILAELAKRLGYGHLYPQTEE-ELLRHVLQGSGYTLEEVKAVG--------------GEVR  539
                        ::  ** *:*    :      :        :       :

DMSOreductase           FPITEGANFVRYADFREDPLF-----NPLGTPSGLIEIYSKNIEKMGYDDCPAHPTWMEP  666
AcetyleneHydratase      ------------MSKLP-DYFGPE-GVGVATPSGKVELYSSVFEKLGYDPLPYYHEPLQT  586
hvMES                   LP----TPMMEYKKYEK-GSLRADGKPGFETPTGKFEIWSSKLEEYGYEPLPKYVEPVEG  583
EaMES                   SP----AAMMEYRKWEK-GLLRPDGRPGFDTPTGKFEIYSTILEEYGYDPLPVYTEPGES  584
CaMES                   KP----TSILEYKKWEK-GLLRSDGQKGFDTPSGKFELASQFLAEHGYDPLPVYTEPTEG  585
AbMES                   LP----AVMMQYKKWMK-GLLRPDGKPGFNTPSGKFEIASSILAEHGYDALPVYVEPGEG  594
                             .      :       . **:* .*:  *  : : **:  * :    :
                               641
DMSOreductase           AERLGGAGAKYPLHVVAS-HPKSRLHSQLNGTSLRDLYAVAGHEPCLINPADAAARGIAD  725
AcetyleneHydratase      EISDPELAKEYPLILFAGLREDSNFQSCYHQPG--ILRDAEPDPVALLHPKTAQSLGLPS  644
hvMES                   PQAAPELSQRFPLVVFNSGARPQTDFRSQHHGIK--GLNKDNPEPTVEINSDDARERGIEP  641
EaMES                   PLSQPEQADRFPLVVFNSGARVTTDFHAQHHSIP--SLVRERPEPTVTLNSHDARERGIRD  642
CaMES                   PLSRPDLAKEFPLVVFNSGSRVTTDFRSQHHGIP--ALNKRRREPQVTMNSADAGERGIKS  643
AbMES                   PLANPQLAADYPLVVFNSGARVFSDFRSQHHGVS--GLREMTPDPIVTMNAVDASERGIEN  652
                               .  :** . :. :  : :::  :      *     .    ::   * s  *:
                                                      715                     737
DMSOreductase           GDVLRVFNDRGQILVGAKVSDAVMPGAIQIYEGGWYDPLDPSEEG----TLDKYGDVNVL  781
AcetyleneHydratase      GEWIWVETTHGRLKLLLKHDGAQPEGTIRIPHGRWCPEQEGGPETGFSGAMLHNDAMVLS  704
hvMES                   GDLVELRTPRGAVPFRARVSQDIVRGAIEANMGGGTPVGPKAW---------QEWNVNEL  692
EaMES                   GDRVIVRTARGEIVLRALVTDGIVRGAVEANMGGGCHQAPEAW---------REGNVNEL  693
CaMES                   GDWVRISTRRGSVVMRAYVTDDIVAGSIDADMGGGGPVGPQAW---------QKCNINDV  694
AbMES                   GDWVNVTTKRGQVKFRAIVTDDIMPGAIDACMGGGGPLGSRSW---------QECNVNEL  703
                        *: : : . :* : .          *::    *        .          :   :
                               755
DMSOreductase           SLDVGTSKLA-QGNCGQTILADVEKYAGAPV--TVTVFDTPKGA----------------  822
AcetyleneHydratase      DDDWNLDPEQGLPNLRGGILAKAYKC----------------------------------  730
hvMES                   TDLYNYDEISGFPVYKALL-CEVVKIKSADAKTRLRLTDSEKGTKADFTFKGSDRKKNRQ  751
EaMES                   TDLARYDPISGFPVYKALL-CDVARAEGDGGRVAIGTG-------EVDSVAGAAPPPEAP  745
CaMES                   TDLQNYDPISGFPVYKTLL-CQVSKVESGKDGTVLDSGE-ASGTIADNGNGRAAQPEFK  752
AbMES                   TDLTRYDPISGFPVYKSLL-AEVVKAGVAKSADRITES-----VPAKTKSRERHRPPKLR  757
```

Figure S2. Top*:* schematic representation of bis-MGD binding in the DMSO reductase.[2] **Bottom***:* Sequence alignment of DMSO reducase, acetylene hydratase and the MPT-binding domains of MES synthases. Conserved residues and conservative substitutions are highlighted in green (relative to DMSOR), semi-conserved substitutions are highlighted in yellow, non-conserved substitutions are highlighted in red. Conserved residues interacting with the ribose and the guanine are shown in the frame; Cys153 in MES is highlighted in blue; four conserved Cys residues involved in the formation of iron/sulfur cluster.

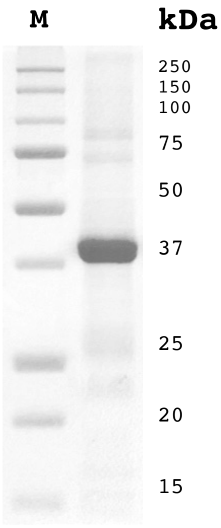

Figure S3. SDS PAGE of purified *Ca*MES$_{\text{C-term}}$. M – molecular weight marker.

Desulfurase activity of CaMES$_{C\text{-}term}$

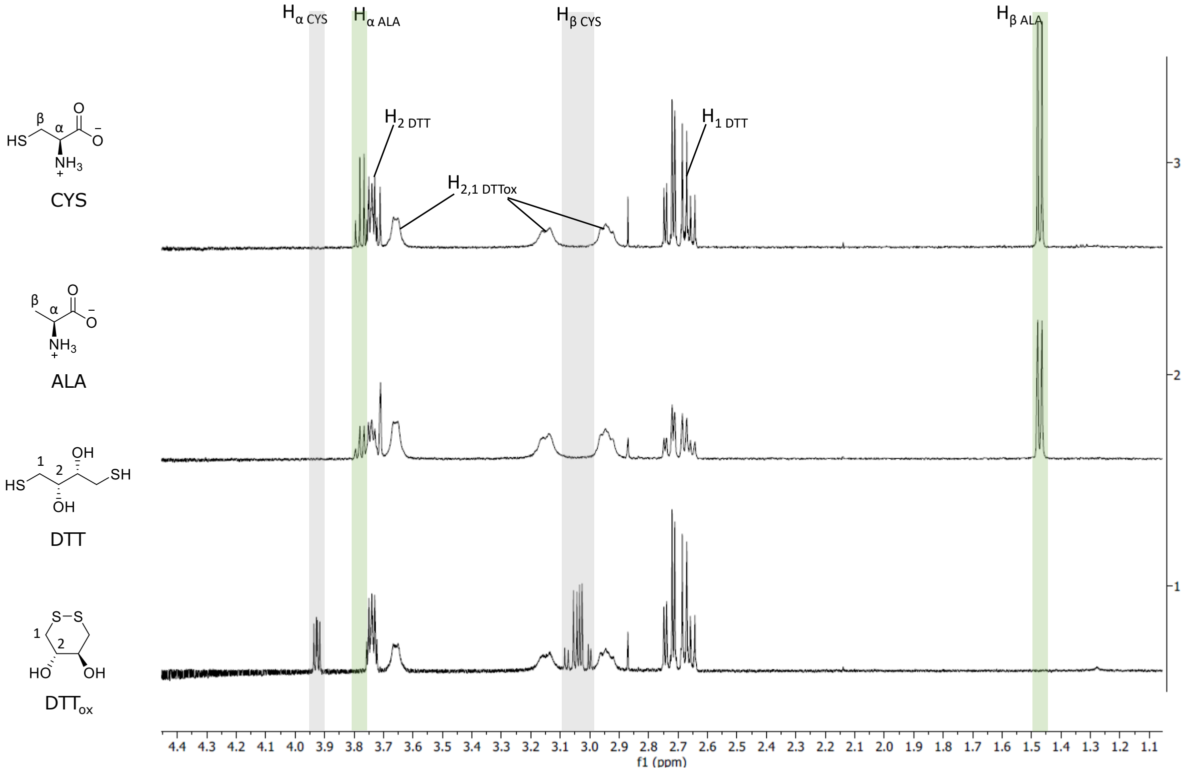

Figure S4. The product of cysteine consumption catalyzed by *Ca*MES$_{C\text{-term}}$, was examined by ^{1}H-NMR (500 Hz, D2O). 3 µM of *Ca*MES$_{C\text{-term}}$ was incubated with 0.5 mM of cysteine, 2 mM of DTT in phosphate buffer (50 mM, pH 8.0) at 25°C overnight. The samples were lyophilized, dissolved in D_2O, and analyzed by ^{1}H-NMR (top spectrum). As control we also analyzed samples containing alanine (the expected product) and DTT (middle spectrum) or cysteine and DTT (bottom spectrum). Comparison of these spectra identifies alanine as the product of cysteine consumption catalyzed by *Ca*MES$_{C\text{-term}}$.

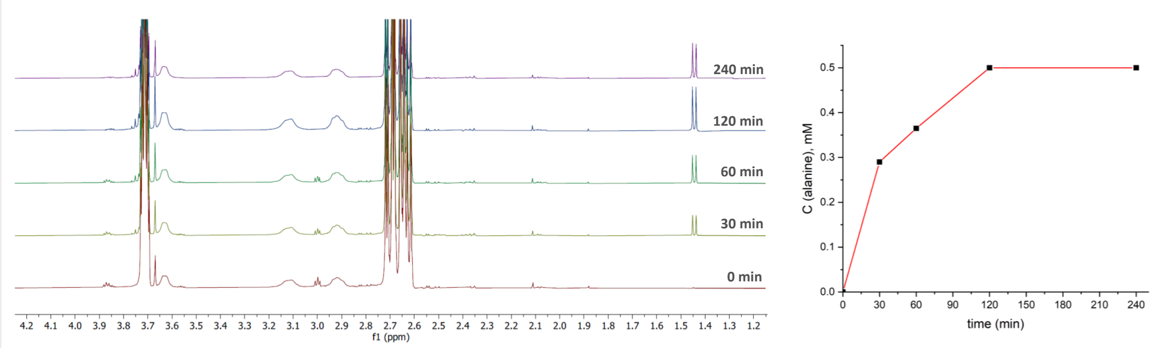

Figure S5. The rate alanine production by *Ca*MES$_{C\text{-term}}$ was quantified as follows: reactions containingof 0.5 mM cysteine, 10 mM DTT, 2 µM of *Ca*MES$_{C\text{-term}}$ in phosphate buffer (50 mM, pH 8.0) were incubated at 25°C for 0, 30, 60, 120, or 240 min. The samples were frozen, lyophilized, dissolved in D_2O, and analyzed by ^{1}H-NMR (left). The concentration of alanine in these reactions was estimated from the integrals of the chemical shifts at 1.45-1.42 ppm. A plot of these values against time shows that *Ca*MES$_{C\text{-term}}$-catalyzed conversion of 0.5 mM cysteine to alanine is complete within 2 h (right)

Characterization of full-length MES in recombinant *E. coli*

Table S2. Growing media recipes.

Luria-Bertani (LB) medium, per 100 mL of H_2O, autoclaved:
1 g tryptone
0.5 g yeast extract
1 g NaCl
Chemically defined (CD) medium, per 100 mL of H_2O:
20 mL 5X M9 salts
5X M9: 34 g/L Na_2HPO_4, 15 g/L KH_2PO_4, 2.5 g/L NaCl, 5.0 g/L NH_4Cl, autoclaved
2 mL 50X mixture of amino acids
50X amino acids: 10g/L each amino acid, sterile filtered
(Met and Tyr are added separately, 20 mg dissolved in water and sterile filtered)
1 mL 40% glucose, autoclaved
100 µL 2M $MgSO_4$, autoclaved
100 µL 100 mM $CaCl_2$, autoclaved
1 mL 100X MEM vitamin solution (Pan Biotech), sterile
sterile H_2O to 100 mL

Table S3. Calculated and observed *m/z* values of ergothioneine.

**compound**	**short name**	***m/z*, calculated**	***m/z*, observed**
Ergothioneine	ERG	230.0958	230.0958
F-ergothioneine	F-ERG	248.0864	248.0861
^{34}S-ergothionene	S-ERG	232.0916	232.0916
D_4-$^{15}N_3$-ergothioneine	DN-ERG (“*heavy*”)	237.1120	237.1123
D_4-$^{15}N_3$-^{34}S-ergothioneine	DNS-ERG (“*super heavy*”)	239.1078	239.1078
$^{13}C_6$-$^{15}N_3$-ergothionene	CN-ERG	239.1071	239.1063
D_6-ergothioneine	D-ERG	236.1340	236.1335
trimethylhistidine	TMH	198.1238	198.1239
Nα-F-trimethylhistidine	F-TMH	216.1143	216.1141
D_5-$^{15}N_3$- trimethylhistidine	DN-TMH	206.1462	206.1460

.

Characterization of MES activity

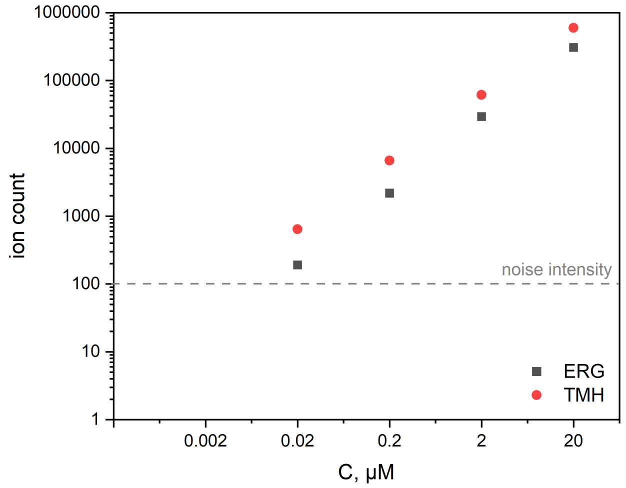

Figure S6. The calibration curve and detection limit of ergothioneine (ERG) and trimethylhistidine (TMH) by RP-HPLC ESI-HR-MS. ERG and TMH concentrations varied between 2 nM to 20 µM. The detection limit is determined as 20 nM.

Table S4. Concentration of ergothioneine and TMH in growth media, additives and untransformed E. coli.[a]

entry		ERG content	TMH content
1	LB, 1 X	< 20 nM	333 ± 21 nM
2	CD, 1 X	< 20 nM	< 20 nM
3	Vitamin mix, 1 X	< 20 nM	< 20 nM
4	Amino acids mix, 1 X	< 20 nM	< 20 nM
5	*E. coli* grown in LB medium	< 20 nM	110 ± 20 nM
6	*E. coli* grown in CD medium	< 20 nM	67 ± 6 nM

[a] Concentrations of ergothioneine and TMH were estimated by HR-ESI-MS based on the external calibration. All measurements were done in in triplicates. Analyzed samples were: entry 1) LB medium; 2) chemically defined medium; 3) mixture of added vitamins in 1 x concentration; 4) amino acid mix in 1 x concentration; 5) Cell-free extracts of *E. coli* Δmtn grown in LB medium for 20 h at 25°C. 6) Cell-free extracts of *E. coli* Δmtn grown in CD medium for 20 h at 25°C. These results show that the growth media did not contain detectable amounts of endogenous ergothioneine. *E. coli* cells that do not contain any recombinant ergothioneine biosynthetic enzyme do not accumulate or produce detectable amounts of ergothioneine.

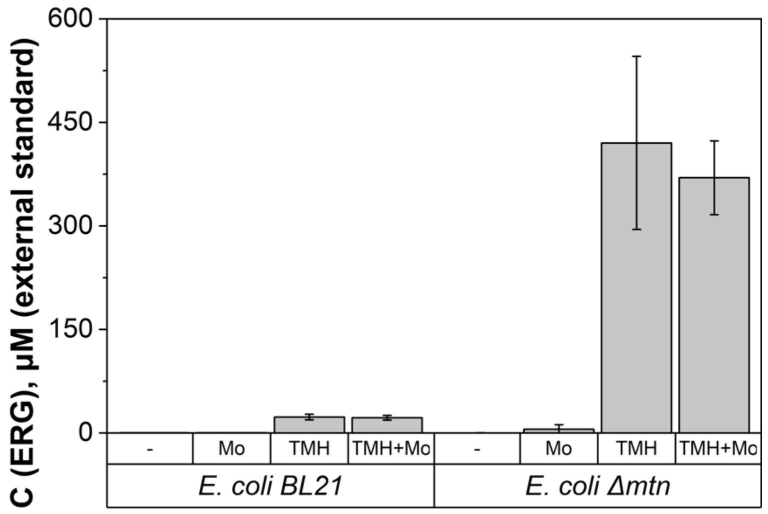

	E. coli **K12 (Δmtn)**		***E. coli*** **BL21**	
	**No metal**	**+ MoO_4^{2-}**	**No metal**	**+ MoO_4^{2-}**
***Ca*MES**	0.08 ± 0.09 µM	5 ± 7 µM	0.13 ± 0.01 µM	0.14 ± 0.02 µM
***Ca*MES + 1 mM TMH**	420 ± 130 µM	370 ± 50 µM	23.0 ± 4 µM	22.0 ± 4 µM

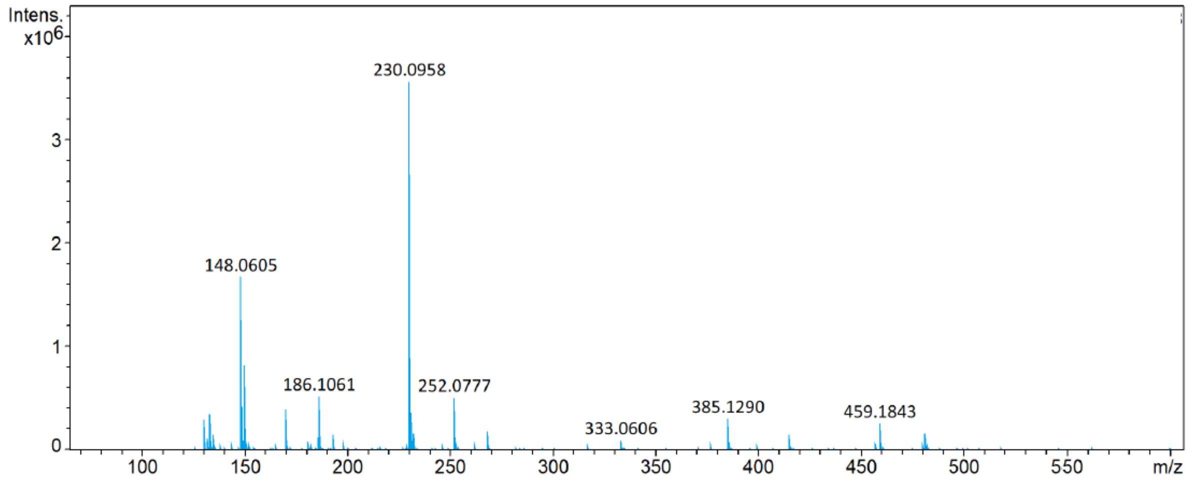

Figure S7. Ergothioneine production by *Ca*MES in *E. coli* BL21 and *E. coli* Δmtn strains grown in CD media supplemented with 10 µM Na_2MoO_4 (+ Mo) and/or 1 mM TMH for 20 h at 25°C. Maximal ergothioneine content in lysate of *E.coli* BL21 cells was estimated as 20 µM; maximal ergothioneine content in lysate of *E. coli* Δmtn cells was estimated as 400 µM (based on external standard). Analysis was done in triplicate and standard deviation is shown. Example of the obtained mass spectra is shown.

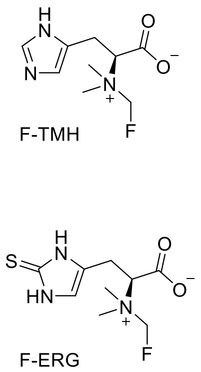

m/z calculated 248.0864
m/z observed 248.0862

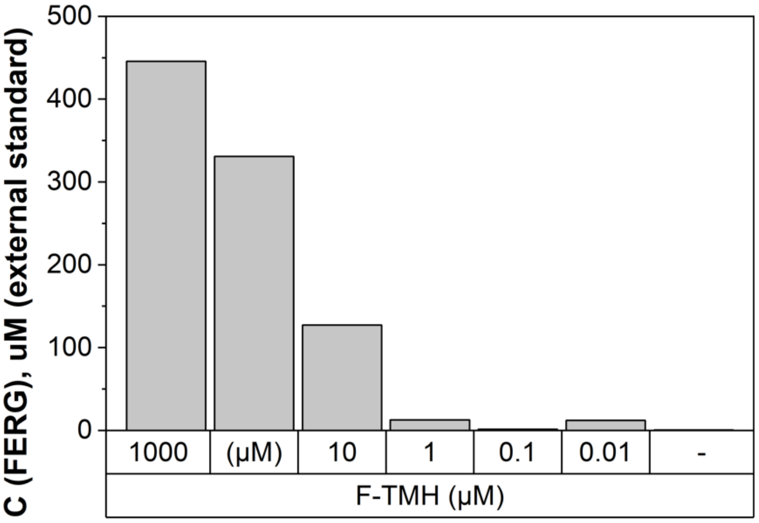

	**C (F-TMH added to media), μM**					
	**-**	**0.1**	**1**	**10**	**100**	**1000**
***Ca*MES + Mo**	0.4	1.2	13	130	330	450

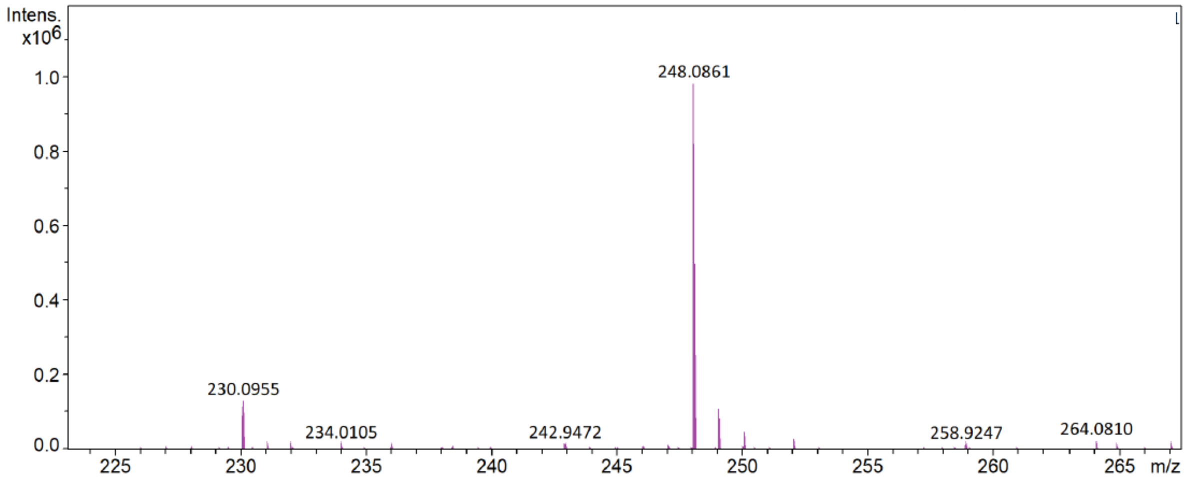

Figure S8. Top left: Structures of *Nα*-methyl fluorinated TMH (F-TMH) and *Nα*-methyl fluorinated ergothioneine (F-ERG). **Top Right:** Production of F-ERG by *Ca*MES from in *E. coli* Δmtn grown in CD media supplemented with 10 μM Na_2MoO_4 and 0.1-μM-1000 μM of F-TMH for 20 h at 25°C. F-ergothioneine content was estimated based on external ergothioneine standard. The signal at 230.0995 corresponds to ergothioneine (produced from endogenous TMH) at a similar level as observed in cells that were not supplemented with TMH (Figure S7). These measurements were done only once. **Bottom:** Example of the obtained mass spectra is shown.

A.

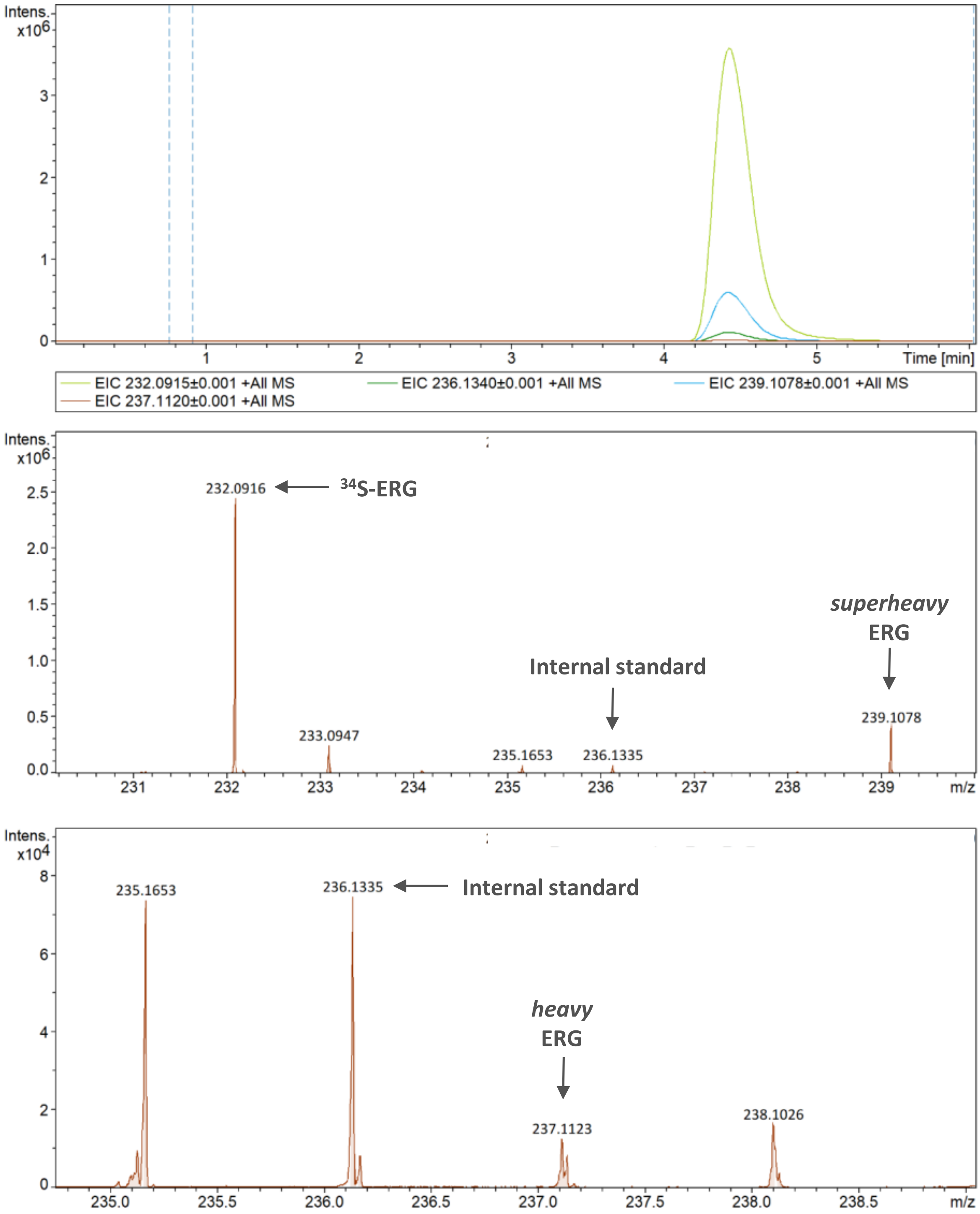

Intens.
x10^6
3
2
1
0
1
2
3
4
5
Time [min]
EIC 232.0915±0.001 +All MS
EIC 236.1340±0.001 +All MS
EIC 239.1078±0.001 +All MS
EIC 237.1120±0.001 +All MS
Intens.
x10^6
2.5
2.0
1.5
1.0
0.5
0.0
232.0916
34S-ERG
233.0947
235.1653
236.1335
Internal standard
superheavy
ERG
239.1078
231
232
233
234
235
236
237
238
239
m/z
Intens.
x10^4
8
6
4
2
0
235.1653
236.1335
Internal standard
heavy
ERG
237.1123
238.1026
235.0
235.5
236.0
236.5
237.0
237.5
238.0
238.5
m/z

B.

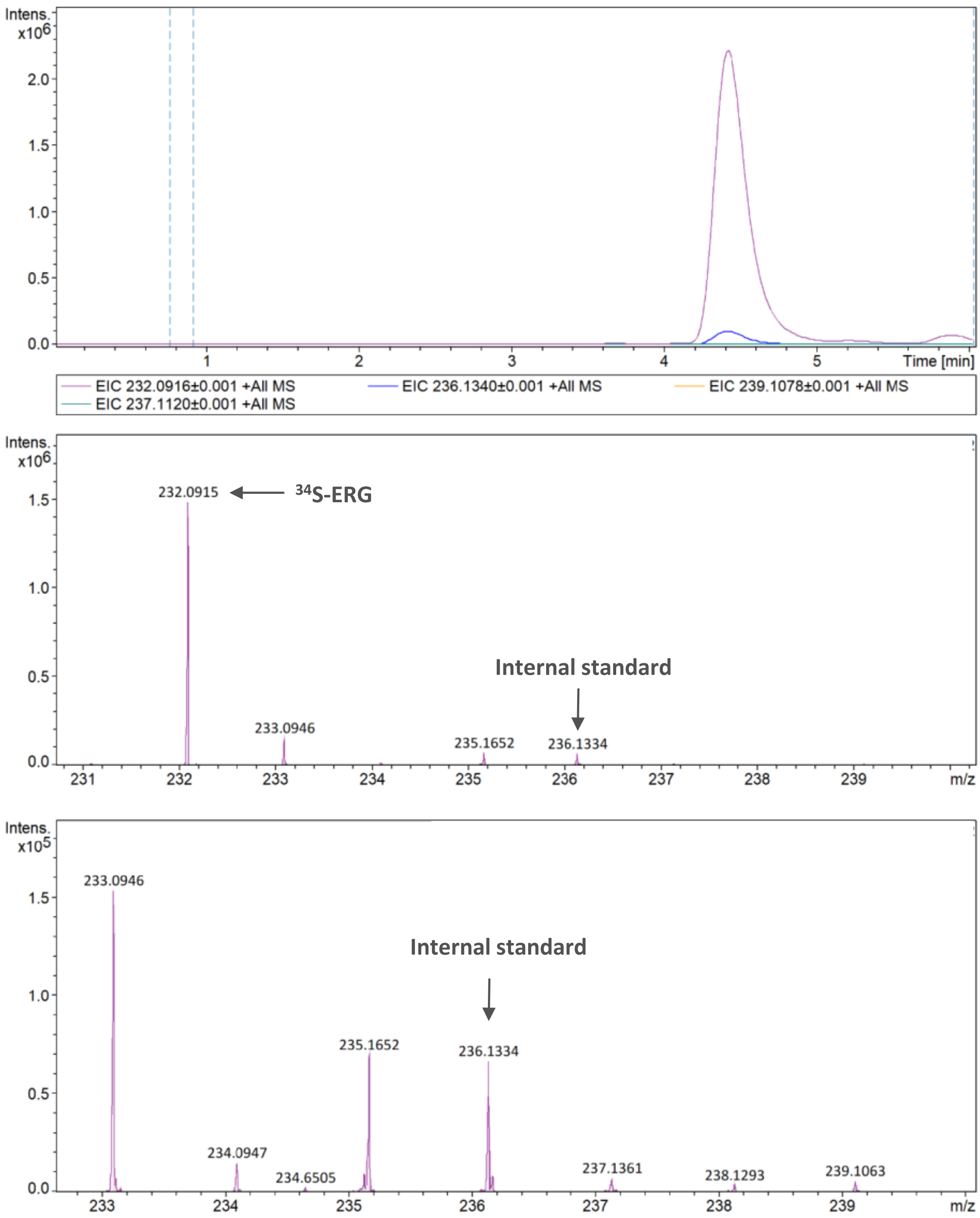

Figure S9. A: ESI-HRMS analysis of *Ca*MES$_{K952A}$ mutant producing *heavy* (m/z calc. 237.1120; obs. 237.1123) and *superheavy* ergothioneine (m/z calc. 239.1078; obs. 239.1076). Top panel – extracted ion chromatogram, middle and bottom panels – average MS spectrum of the peak (t_R 4.1-4.9 min). **B:** Control ESI-HRMS analysis of *Ca*MES$_{C153S}$ mutant unable to produce *heavy* and *superheavy* ergothioneine. The signals at 237.13761 and 239.1063 do not correspond to *heavy* and *superheavy* ergothioneine because these masses are outside the 5 ppm tolerance of the calculated masses (Table S3). Top panel – extracted ion chromatogram, middle and bottom panels – average MS spectrum of the peak (t_R 4.1-4.9 min).

References

1. Vit, A.; Misson, L.; Blankenfeldt, W.; Seebeck, F. P., Ergothioneine biosynthetic methyltransferase EgtD reveals the structural basis of aromatic amino acid betaine biosynthesis. *Chembiochem* **2015,** *16* (1), 119-25.
2. Schindelin, H.; Kisker, C.; Hilton, J.; Rajagopalan, K. V.; Rees, D. C., Crystal Structure of DMSO Reductase: Redox-Linked Changes in Molybdopterin Coordination. *Science* **1996,** *272*, 1615-1621.
